# The economic burden of dementia in low- and middle-income countries (LMICs): a systematic review

**DOI:** 10.1136/bmjgh-2021-007409

**Published:** 2022-04-03

**Authors:** Siti Maisarah Mattap, Devi Mohan, Andrea Mary McGrattan, Pascale Allotey, Blossom CM Stephan, Daniel D Reidpath, Mario Siervo, Louise Robinson, Nathorn Chaiyakunapruk

**Affiliations:** 1 Global Public Health, Jeffrey Cheah School of Medicine and Health Sciences, Monash University Malaysia, Bandar Sunway, Selangor, Malaysia; 2 School of Biomedical, Nutritional and Sports Sciences, Newcastle University, Newcastle upon Tyne, UK; 3 United Nations University International Institute for Global Health, Bandar Tun Razak, Wilayah Persekutuan Kuala Lumpur, Malaysia; 4 Institute of Mental Health, University of Nottingham, Nottingham, UK; 5 International Centre for Diarrhoeal Disease Research Bangladesh, Dhaka, Bangladesh; 6 Jeffrey Cheah School of Medicine and Health Sciences, Monash University Malaysia, Bandar Sunway, Selangor, Malaysia; 7 School of Life Sciences, University of Nottingham, Nottingham, UK; 8 Population Health Sciences Institute, Newcastle University, Newcastle upon Tyne, UK; 9 Department of Pharmacotherapy, The University of Utah College of Pharmacy, Salt Lake City, Utah, USA; 10 School of Pharmacy, Monash University Malaysia, Selangor, Malaysia; 11 IDEAS Center, Veterans Affairs Salt Lake City Healthcare System, Salt Lake City, Utah, USA

**Keywords:** systematic review, health economics, public Health

## Abstract

**Introduction:**

More than two-thirds of people with dementia live in low- and middle-income countries (LMICs), resulting in a significant economic burden in these settings. In this systematic review, we consolidate the existing evidence on the cost of dementia in LMICs.

**Methods:**

Six databases were searched for original research reporting on the costs associated with all-cause dementia or its subtypes in LMICs. The national-level dementia costs inflated to 2019 were expressed as percentages of each country’s gross domestic product (GDP) and summarised as the total mean percentage of GDP. The risk of bias of studies was assessed using the Larg and Moss method.

**Results:**

We identified 14 095 articles, of which 24 studies met the eligibility criteria. Most studies had a low risk of bias. Of the 138 LMICs, data were available from 122 countries. The total annual absolute per capita cost ranged from US$590.78 for mild dementia to US$25 510.66 for severe dementia. Costs increased with the severity of dementia and the number of comorbidities. The estimated annual total national costs of dementia ranged from US$1.04 million in Vanuatu to US$195 billion in China. The average total national expenditure on dementia estimated as a proportion of GDP in LMICs was 0.45%. Indirect costs, on average, accounted for 58% of the total cost of dementia, while direct costs contributed 42%. Lack of nationally representative samples, variation in cost components, and quantification of indirect cost were the major methodological challenges identified in the existing studies.

**Conclusion:**

The estimated costs of dementia in LMICs are lower than in high-income countries. Indirect costs contribute the most to the LMIC cost. Early detection of dementia and management of comorbidities is essential for reducing costs. The current costs are likely to be an underestimation due to limited dementia costing studies conducted in LMICs, especially in countries defined as low- income.

**PROSPERO registration number:**

The protocol was registered in the International Prospective Register of Systematic Reviews database with registration number CRD42020191321.

**WHAT IS ALREADY KNOWN ON THIS TOPIC**

- Previous review and studies on the economic burden of dementia are heavily based on high-income countries, while a larger proportion of people with dementia lives in low- and middle-income countries (LMICs).
- No reviews on the economic burden of dementia or determinants of the cost have been published before.

**WHAT THIS STUDY ADDS**

- This is the first systematic review focused on the economic burden of dementia in LMICs.
- Our review established that in LMICs, indirect cost is the major contributor to the high economic burden of dementia. The costs increase as the severity and number of comorbidities increases.
- Our study findings emphasise that the studies from LMICs faced methodological challenges, especially in the recruitment of participants, standardisation of measured costs items, and indirect cost components.

**HOW THIS STUDY MIGHT AFFECT RESEARCH, PRACTICE AND/OR POLICY**

- Based on our study findings, we recommend that future research studies on the economic burden of dementia in LMICs use standardised methods to measure the direct and indirect costs.
- Inclusion of reduced working time data from patient and caregiver and informal caregiving time outside the reduced work time without double counting in the indirect costing calculation are encouraged.
- Nationally representative samples of dementia patients, including community-dwelling and institutionalised people, are required to allow for comparisons between countries.
- The health systems in LMICs should focus on the slowdown of dementia progression and control of comorbid conditions to reduce the direct cost of dementia in the long run.
- Assistance for caregivers of people living with dementia in these regions should be enhanced to reduce the burden of caregiving, thus, the indirect cost.

## Introduction

Dementia is a syndrome causing deterioration in memory, thinking, behaviour and the physical functioning.^1^ It is estimated that 50 million people are living with dementia globally.^2^ More than two-thirds of them were in low- and middle-income countries (LMICs).^3^ In 2015, the estimated global economic burden of dementia was US$818 billion or 1.09% of aggregated global gross domestic product (GDP).^3^ This figure is projected to reach US$2 trillion by 2030.^2^ However, these estimates are heavily based on studies from high-income countries (HICs). Indeed, only 10% of the studies in this estimation were from LMICs.^2^ As such, current cost of global dementia may not be representative of the LMICs.

Levels of mental functioning and dependency deteriorate as dementia progresses to a severe stage.^4^ Therefore, the cost will vary with disease severity. Existing reviews have shown differences in the direct medical, direct non-medical and indirect costs associated with dementia.^5–8^ Studies from HICs have reported that the indirect costs are higher than direct costs.^9–11^ The main drivers of dementia costs in HICs are home based care and nursing costs.^7^ There is lack of such studies from LMICs, but the costs are expected to be different from HIC due to differences in services, infrastructure and cultural perception of ageing and dementia (eg, disease vs not a disease).^12^ A thorough review of the dementia costs and its contributors in LMICs is also necessary to inform stakeholders to plan the healthcare and social care delivery for people affected by dementia in these countries.

Given the urgency for increased efforts to improve outcomes of dementia in LMICs,^13^ the importance of good-quality economic data is unquestionable. Also, identifying the challenges in estimating the dementia costs specific to LMICs will be crucial to develop methodological recommendations to improve the quality of economic data from these settings. This review aimed to systematically review existing evidence of dementia costs in LMICs and to conduct methodological assessment of the included studies.

## Method

### Search strategy and selection criteria

The systematic review was conducted according to the Preferred Reporting Items for Systematic Reviews and Meta-Analyses (PRISMA).^14^ A comprehensive search in six electronic databases (EconLit, EMBASE, PubMed, Cochrane Review (DARE), ERIC and PsycINFO) was performed. The search strategy was based on three broad search strings (‘cost of illness’) AND (‘Dementia’ OR ‘AD*’) AND (LMICs). The complete search strategy can be found in online supplemental material pages 1-6. Potentially relevant publications were retrieved from the databases from inception to September 2020, without language restriction. Additional literature was identified by snowballing the reference list of the eligible studies and via a grey literature search of government and international agencies reports.

10.1136/bmjgh-2021-007409.supp1 Supplementary data

### Study selection

We included studies based on three criteria: original research article, reported the economic burden for all-cause dementia and/or its types, and the study setting was in LMICs. The search included 138 LMICs according to the World Bank in 2020^15^. Quantitative studies which reported any cost of dementia, including direct medical, direct non-medical and indirect costs were included. We did not assess the intangible cost as it was challenging to quantify their monetary value. Studies on economic evaluation of specific dementia interventions, editorials, animal studies, reviews, case studies and case series were excluded. Selection criteria were not limited to any specific language, thus minimising language bias.

**Figure 1:**
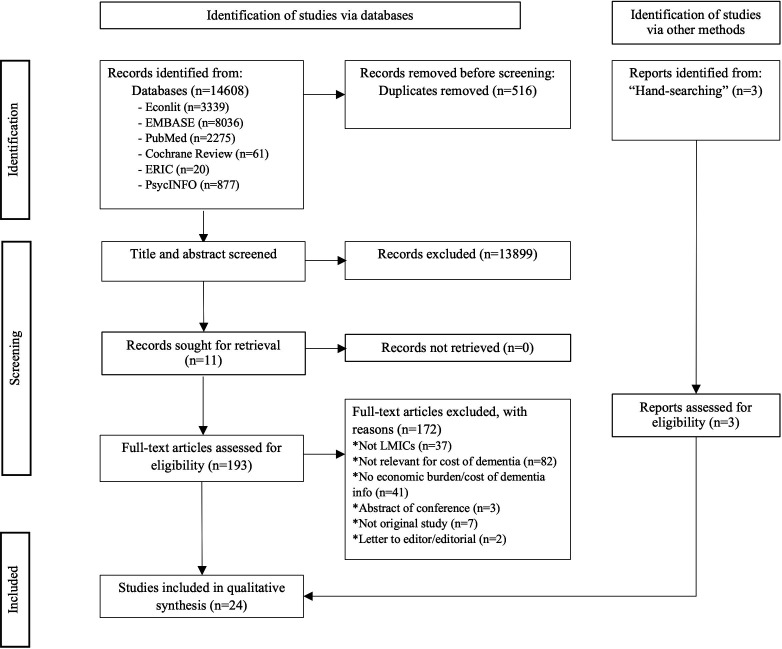
PRISMA flow chart. LMICs, low- and middle-income countries; PRISMA, Preferred Reporting Items for Systematic Reviews and Meta-Analyses.

Two reviewers (SMM and AMM) independently screened the title and abstract, followed by full-text screening. Disagreements were resolved by consensus or by consulting a third reviewer (DM). The PRISMA flow chart illustrating the screening process is shown in figure 1. For studies in which the price year was not reported, the publication year was used. One reviewer (SMM) collected data from each report, and the research team confirmed data validity.

### Data analysis

Data on author(s), publication year, type of dementia, study design, aims, participants (sample size, mean age, gender), economic components, data sources, cost unit(s), estimates of total costs, currency, comorbidities reported, price year and key findings were extracted. The risk of study bias was assessed using the Larg and Moss method^16^ as shown in online supplemental table 1.

Data were synthesised under the following domains- study characteristics, key findings, studies’ methodological quality and estimation of dementia costs. All costs were inflated from the reported year to 2019 values using country-specific Consumer Price Index (CPI) data from the World Bank^17^ and subsequently converted to US dollars (US$) according to the recommendations of Turner 2019.^18^ The most updated data were used in the final cost summary measures for studies estimating cost from the same data source at different time points.

To further facilitate comparison of dementia costs at the national level across countries, we estimated the cost of dementia, as a percentage of the country’s GDP in 2019. Unweighted means and means weighted by population (to account for the differences in population size) were calculated. Data on population size, CPI, exchange rate and GDP were obtained from the World Bank website^19^ and, if unavailable, were taken from the International Monetary Fund^20^ and other appropriate available sources listed in the online supplemental table 2.

### Patient and public involvement

Patients were not involved in the design or conduct of this systematic review.

## Results

### Study characteristics

We identified 14 095 studies after removing duplicates. After title and abstract screening, 196 references were selected for full-paper screening. Of these, 172 articles were excluded (as shown in figure 1) based on the inclusion and exclusion criteria, including two near-missed articles. One of the near misses was an editorial with a brief estimation of Pakistan’s dementia cost using data from India in 10/66 Dementia study.^21^ The second one was a letter to the editor with an estimation of dementia cost in Nigeria;^22^ both of the articles were not published elsewhere. In total, 22 study articles^3 23–43^ and 2 published theses^30 44^ were selected for inclusion.

**Figure 2:**
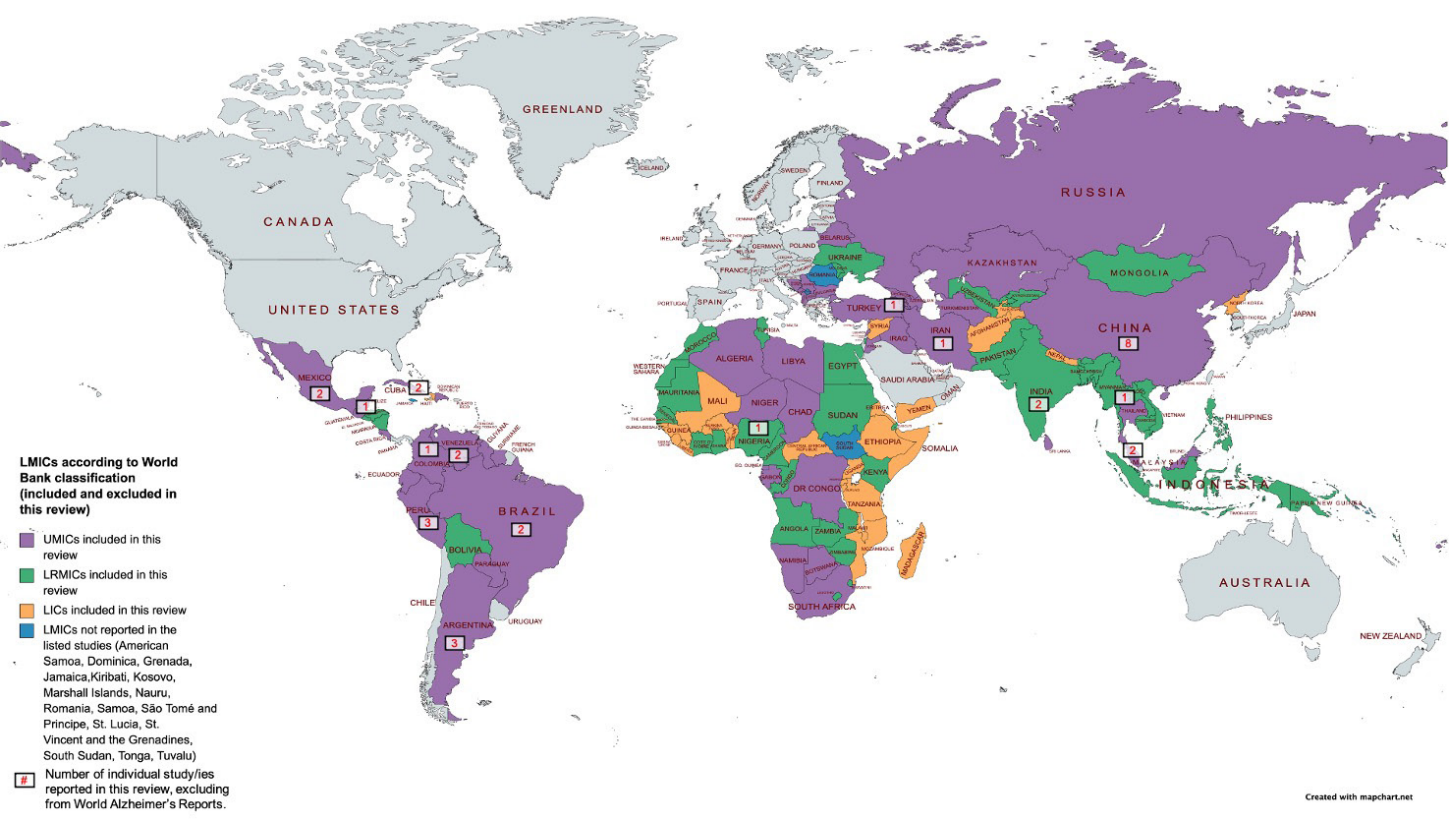
World map depicting study site included and excluded in this review based on World Bank countries’ income classification, and number of studies in each site excluding the World AD’s Reports. LICs, low-income countries; LMICs, low- and middle-income countries; LRMICs, lower-middle-income countries; UMICs, upper-middle-income countries.

Dementia costs from 122 LMICs (out of 138) were reported within the included studies as shown in figure 2. Table 1 summarises the key characteristics of the 24 included studies. The studies were categorised based on the cost estimation sources. The first group includes 16 studies^23–26 28–31 33 36–39 44–46^ using collected original data, the second group consists of 6 studies^3 27 40–43^ using prevalence estimates of dementia, and the third group includes two studies^32 35^ using a combination of original data and prevalence estimates. The first group reported patient-level cost of dementia, except in two studies^38 44^ which showed both patient-level and total national expenditure estimate, and one study^30^ showed dementia cost as per episode of care. In contrast, all the second and third groups of studies,^3 27 32 35 40–43^ showed patient level and total national expenditure estimate, except one study^27^ that measured change in net present value per person and predicted China’s dementia burden from 2011 to 2050.

**Table 1:** Characteristic of studies included in this systematic review

Author, year	Country	World bank classification	Dementia type	Dementia diagnosis	Perspective	Sample characteristics	Costing approach/method	Data source	Costing level	Reported cost			Comorbidities assessed in the cost analysis
										Direct medical	Direct Non-medical	Indirect	
Aajami *et al*, 2019^23^	Iran	UMIC	AD	Clinical diagnosis	Societal	Sample size: 300 patients, 300 caregivers, no controls. Any AD severity level. Mean age=80 (4.56), 52% females; only urban sample; AD society.	Descriptive cost; patient level primary data	Interview and Medical records	Annual cost per person	✓	✓	✓	No
Allegri *et al*, 2007^24^	Argentina	UMIC	AD	NINCDS-ADRDA	Societal	Sample size: 100 patients, 100 caregivers, 25 controls. Any AD severity level. Mean age=74.58 (7.76); 56% females; only urban sample; mixed community and institutionalised.	Descriptive cost; patient level primary data	Interview	Annual cost per person	✓	✓	✓	Yes
Custodio *et al,* 2015^25^	Peru	UMIC	AD, FTD and VaD	DSM-IV-TR, NINCDS-ADRDA, Lund and Manchester criteria, NINDS AIREN criteria	HC payer	Sample size: 106 patients, 106 caregivers, 30 controls. Any dementia severity level. Mean age=70.01 (4.83), 59% females; only urban sample; single clinic based	Descriptive cost; patient level primary data	Interview and Medical records	3 months cost per person	✓	✓	✓	No
Ferretti *et al,* 2018^26^	Brazil	UMIC	Dementia	Doctor/nurse diagnosis	Societal	Sample size: 156 patients, 156 caregivers, no controls. Any dementia severity level. Mean age=72.9 (10.2), 58% females; only urban sample; single hospital based	Descriptive cost; patient level primary data	Interview	Monthly cost per person	✓	✓	✓	Yes
Jia *et al*, 2018^38^	China	UMIC	AD	NINCDS-ADRDA	Societal	Sample size: 3046 patients, 3046 caregivers, no controls. Any AD severity level. Mean age=70–79*, 54% females; urban and rural sample; multicentre and cluster randomised.	Descriptive analysis and prevalence-based estimation; patient level primary data and national level assumption.	Interview and Medical records	Annual cost per person and annual total country’s expenditure	✓	✓	✓	Yes
Keogh-Brown *et al*, 2016^27^†	China	UMIC	AD	NA	Societal	NA	Multistate probabilistic simulation model of AD progression; national level data	Prevalence based	Annual change in net present value GDP per person	✓	✓	✓	No
Kongpakwattana *et al*, 2019^28^	Thailand	UMIC	AD	Doctor diagnosis	Societal	Sample size: 148 patients, 148 caregivers, no controls. Any AD severity level. Mean age=80.1 (8.0), 71% females; only urban sample; mixed community and nursing institution based.	Descriptive cost; patient level primary data	Interview	Annual cost per person	✓	✓	✓	Yes
Koris, 2018^44^‡	Malaysia	UMIC	Dementia	Doctor diagnosis	Societal	Sample size: 404 patients, caregivers and controls not specified. Mode age =70–79*; 71% females; urban and rural sample; single hospital based.	Descriptive cost; patient and national level primary data	Interview and Medical records	Annual cost per person and annual total country’s expenditure	✓	✓	✓	Yes
Liu, 2013^45^‡	China, Cuba, Dominican Republic, India, Mexico, Peru, Venezuela	UMIC, LRMIC	Dementia	Validated 10/66 dementia diagnostic algorithm and/or DSM IV dementia criteria	Societal	Sample size: 1379 patients, 15 022 caregiver, 13 643 controls. Any dementia severity level. Mode age ≥80 * 67% females; urban and rural sample; single hospital based.	Descriptive cost; patient level primary data	Interview and Survey	Annual cost per person	✓	✓	✓	Yes
Mould-Quevedo *et al*, 2013^29^	China	UMIC	Dementia	DSM-IV-TR, NINCDS-ADRDA	HC payer	Sample size: 1387 patients, 1387 caregivers, no controls. Any dementia severity level. Mean age=67.6 (12.8); 48% females; urban and rural sample; randomised multicentre hospitals and nursing institutions based.	Descriptive cost; patient level primary data	Interview	Monthly cost per person	✓	✓	✓	No
Nur *et al*, 2017^30^	Malaysia	UMIC	Dementia	Doctor diagnosis	HC provider	Sample size: 142 patients, no caregivers, no controls. Any dementia severity level. Mean age=75 (8.05); 50% females; urban and rural sample; randomised multicentre hospital based.	Descriptive cost; patient level primary data	Medical records	Per episode of care per person	✓	✗	✗	No
Prada *et al,* 2017^39^	Colombia	UMIC	Dementia	ICD-10	HC provider	Sample size: 340 patients, no caregivers, no controls. Any dementia severity level. Mode age >50*; 57% females; urban and rural sample; insurance claim database.	Descriptive cost; patient level primary data	Insurer database	Annual cost per person	✓	✗	✗	Yes
Prince *et al*, 2004^47^	Argentina, Brazil, Chile, Cuba, Dominican Republic, Guatemala, Mexico, Panama, Peru, Uruguay, Venezuela, China, India, Nigeria	HIC, UMIC, LRMIC	Dementia	DSM-IV	Societal	Sample size: 706 patients, 706 caregivers, no control. Severe dementia excluded. Mean age=NA; 53% females; urban and rural sample; multi-centre community based.	Descriptive cost; patient level primary data	Interview and Client Service Receipt Inventory	Monthly cost per person	✓	✓	✓	No
Rao and Bharath, 2013^32^	India	LRMIC	Dementia	DSM-IV/ICD 10	Societal	NA	Preavalence based estimation; patient level primary data and national level assumption.	Medical records and cost assumption	Annual cost per person and annual total country’s expenditure	✓	✓	✓	No
Rojas *et al*, 2011^33^	Argentina	UMIC	AD, FTD and VaD	DSM-IV-TR, NINCDS-ADRDA, Lund and Manchester criteria, NINDS AIREN criteria	HC payer	Sample size: 104 patients, 104 caregivers, 29 controls. Any dementia severity level. Mean age=68.8 (6.62); 53% females; only urban sample; single hospital based.	Descriptive cost; patient level primary data	Interview	Annual cost per person	✓	✓	✗	No
Wang *et al,* 2008^34^	China	UMIC	AD	DSM-IV-TR	Societal	Sample size: 66 patients, 66 caregivers, no control. Any AD severity level. Mean age=74 (53–90), gender not specified; urban sample; single hospital based.	Descriptive cost; patient level primary data	Interview	Annual cost per person	✓	✓	✓	No
Wimo *et al,* 2006^40^	Worldwide	HIC, UMIC, LRMIC, LIC	Dementia	NA	HC payer	NA	Prevalence-based estimation; national level assumption.	Prevalence based	Annual aggregated cost by major regions	✓	✓	✗	No
Wimo *et al,* 2007^42^	Worldwide	HIC, UMIC, LRMIC, LIC	Dementia	NA	Societal	NA	Prevalence-based estimation; national level assumption.	Prevalence based	Annual aggregated cost by major world regions and country	✓	✓	✓	No
Wimo *et al*, 2010^43^	Worldwide	HIC, UMIC, LRMIC, LIC	Dementia	NA	Societal	NA	Prevalence-based estimation; national level assumption.	Prevalence based	Annual aggregated cost by major world regions and country	✓	✓	✓	No
Wimo *et al,* 2013^40^	Worldwide	HIC, UMIC, LRMIC, LIC	Dementia	NA	Societal	NA	Prevalence-based estimation; national level assumption.	Prevalence based	Annual aggregated and per person cost by major world regions	✓	✓	✓	No
Wimo *et al,* 2017^3^	Worldwide	HIC, UMIC, LRMIC, LIC	Dementia	Validated 10/66 dementia diagnostic algorithm and/or DSM IV dementia criteria (for LMIC from Liu, 2013)^45^	Societal	NA	Prevalence-based estimation; national level assumption.	Prevalence-based	Annual aggregated and per person cost by major world regions	✓	✓	✓	No
Xu *et al*, 2017^35^	China	UMIC	Dementia	ICD-10	Societal	Sample size: 146 patients, 66 caregivers, no control. Any dementia severity level. Mode age =75–79* | 62% females; urban and rural sample; two hospitals based.	Descriptive cost and prevalence-based estimation; national level assumption.	Medical records and Prevalence based	Annual cost per person and annual total country’s expenditure	✓	✓	✓	No
Yan *et al,* 2019^36^	China	UMIC	AD	NINCDS-ADRDA	Societal	Sample size: 3046 patients, 8041.4 caregivers, no control. Any AD severity level. Mean age=75.27 (9.4); 54% females; urban and rural sample; multicentre hospitals and institutions based.	Descriptive cost; patient level primary data	Interview and Medical records	Annual cost per person	✓	✓	✓	Yes
Zencir *et al*, 2005^37^	Turkey	UMIC	AD	DSM-IV	Societal	Sample size: 42 patients, 42 caregivers, no control. Any AD severity level. 70.5 (8.9) | 62% females; only urban sample; single AD association based.	Descriptive cost; patient level primary data	Interview	Annual cost per person	✓	✗	✓	No

*Mode.

†Keogh-Brown study is not a traditional cost-of-illness study.

‡From thesis publication.

AD, Alzheimer’s disease; ANOVA, Analysis of variance; CDR, Clinical Dementia Rating; DSM-IV-TR, Diagnostic and Statistical Manual of Mental Disorders, Fourth Edition criteria; FAST scale, Functional Assessment Staging Scale; FTD, frontotemporal dementia; GDP, gross domestic product; GLM, generalised linear model; HC, healthcare; HIC, high-income country; ICD-10, International Classification of Diseases 10th Revision; LIC, low-income countries; LRMICs, lower-middle-income countries; NA, not available; NINCDS-ADRDA, National Institute of Neurological and Communicative Disorders and Stroke and Alzheimer’s Disease and Related Disorders Association criteria; NINDS AIREN, National Institute of Neurological Disorders and Stroke and the Association Internationale pour la Recherche et l’Enseignement en Neurosciences; UMICs, upper-middle-income countries; VaD, Vascular Dementia.

Out of all studies, 7 studies^3 31 40–43 45^ reported combination of LMICs (five studies were worldwide economic burden from World AD Report (WAR) released by the AD’s Association and 2 from 10/66 Dementia Group study), 16 studies^23–30 33 35–39 44 46^ were from upper-middle-income countries (UMICs) and 1 study^32^ from lower-middle-income countries (LRMICs). The studies from WAR^3 40–43^ are the most comprehensive cost estimation report of the worldwide cost of dementia, but contribution from LMICs countries’ original data within these reports is lacking; thus costs imputations were done for the countries with no data. Fourteen studies^3 26 29–32 35 39–45^ assessed the cost of all-cause dementia, while eight studies^23 24 27 28 36–38 46^ assessed the cost of AD and two studies^25 33^ assessed the cost of cause-specific dementia.

Most studies (n=18^3 23 24 26–28 31 32 35–38 40–45^) costed dementia from a societal perspective, while four studies^25 29 33 41^ used a healthcare payer perspective, and two studies^30 39^ used a healthcare provider perspective. The most common study design was cross-sectional (n=16^23–26 28–33 36–38 44–46^), followed by five studies with worldwide cost estimation report from WAR,^3 40–43^ two longitudinal,^35 39^ and one simulation modelling^27^ (not shown in table 1). Study data were frequently sourced from the patient’s medical records and/or patient/carer interviews (n=16^23–26 28–31 33 35–38 44–46^), followed by national prevalence based estimates (n=6^3 27 40–43^), insurance claim data (n=1^39^), and a combination of medical records and cost assumption based on dementia governing body (n=1^32^). Although comorbidities are major issues among for older dementia patients, only eight studies^24 26 28 36 38 39 44 45^ assessed comorbidities either in descriptively or as a determinant of dementia costs.

Of the 18 studies that collected patient-level primary data, 14 studies^23–26 28 29 31 33 35–38 45 46^ recruited both patients and informal caregivers; however, 2 studies^32 44^ did not specify the caregiver information, and two studies^30 33^ did not include caregivers (due to data from hospital medical records or insurer database). The study participants in all the 18 studies were aged 60-year-old and above and with any severity of dementia except for one study^31^ that excluded severe dementia. Most studies (n=11)^27 29–32 35 36 38 39 44 45^ included patients from rural and urban locations. Four studies^24 25 33 45^ with patient-level data had recruited control samples.

### Risk of bias and study methodology assessment

The full assessment of the risk of bias is shown in the online supplemental table 1. In general, most studies exhibited a low risk of bias in the analytical framework and methodology sections, while moderate risk was observed in the analysis and reporting section of the Larg and Moss framework.

In the analytical framework component, all studies exhibited low risk except for two studies^29 37^ in which identification of the non-trivial cost components were limited. Zencir *et al*^37^ did not report hospitalisation and formal care costs but were acknowledged in the study limitation section. Meanwhile, Mould-Quevedo *et al*^29^ did not report the direct non-medical cost and indirect cost in monetary value even though it is a societal perspective study and misclassified paid caregiver under the indirect cost category.

Low risk of bias was observed in the methodology and data section except in three areas. First, most studies (n=22^3 23–33 35–38 40–44 46^) did not measure additional or excess costs due to dementia. Second, the data sampling by nine studies^26 28 29 31 33 37 39 45 46^ was not representative of the study population stated in the aim. Out of 18 studies conducted using primary data, only four studies^30 36 38 44^ obtained their data through random sampling, while the remaining studies used convenient sampling. However, all 18 studies include all dementia severity levels, except one study^31^ in which severe dementia patients were not represented. Third, two studies^24 33^ included cost components unrelated or not specific to dementia cost (ie, ancillary study).

A moderate risk of bias was identified in the analysis and reporting section. Twelve^3 24 27 31 32 35 38–43^ out of 24 studies did not provide the range of cost estimates. Ten studies did not report any sensitivity analysis.^23 26 29–32 37 43 44 46^ Ten studies^3 24 25 27 28 35 40–42 45^ reported sensitivity analysis on the important parameter estimates and key assumptions, while four studies^33 36 38 39^ performed sensitivity analysis only on the important parameter estimates. None of the studies performed a sensitivity analysis on the point estimates.

Two methodological criteria were assessed in detail: quality of healthcare resources and indirect cost estimation. The quality of healthcare resources evaluation and quantification were done appropriately in most studies. However, variations in the individual cost components were observed as shown in online supplemental tables 3-5.

All studies reported direct medical cost, but only 19 listed the individual cost components. The studies from WAR^3 40–43^ did not separately report the direct medical and direct non-medical cost components. Important direct cost components that were not reported in some studies were medications (n=7^3 31 33 40–43^), hospital stays (n=3^23 25 37^), and outpatients visits cost (n=2^31 45^)

One of the major challenges regarding healthcare quantification of direct cost is whether the data were collected from private or public health facilities. Ten studies^24–27 30 31 33 39 44 45^ collected data from private (n=2^25 39^) or public (n=2^30 33^) or both (n=4^24 26 27 31^), including two studies^44 45^ which quantified private and public direct cost separately. These two studies^44 45^ showed patients who went to private healthcare had a higher cost than those who went to public healthcare providers.

Direct non-medical cost was included in 21 studies.^3 23–29 31–33 35 36 38 40–46^ Sixteen studies^23–29 31–33 35 36 38 44–46^ reported individual components including transportation (n=11^23 26–28 32 35 36 38 44–46^), paid caregivers (n=9^24 25 27 28 31–33 44 46^), nursing home or institutionalisation (n=6^23 24 32 35 36 38^), meals or special diet (n=5^23 32 36 38 44^), special equipment (n=4^23 27 36 46^), and nappy (n=2^25 26^)

Indirect costs components included productivity loss (income loss) of the caregiver and/or patient and informal caregiver time and were assessed in 20 studies.^3 23–29 31 32 35–38 40 42–46^ Most studies utilised an opportunity costing method (n=12^3 26 28 31 32 35 36 38 40 42–44^) compared with a replacement cost method (n=5^23–25 37 46^) to estimate caregiving burden. One study^45^ used the human capital approach to value productivity loss, and two studies^27 29^ did not report their indirect costing method.

Out of 20 studies assessing indirect cost, caregiving time was assessed in 15 studies,^3 24–28 32 35 37 40 42–46^ productivity work loss of informal caregiver was included in 10 studies,^23 26 27 29 31 32 35–38^ and productivity work loss of patient was included in 3 studies.^35 36 38^ Rarely, health services for caregiver treatment were included (n=3^26 36 38^).

### Cost of dementia in LMICs

The economic burden of dementia was presented in the included studies in various ways. While all studies reported the cost of dementia as absolute cost, two studies included the attributable cost of dementia.^24 45^ The cost of dementia was also presented using the catastrophic health expenditure in one study.^44^ The dementia costs estimation were reported as per person/per capita expenditure (n= 13^23–26 28 29 31 33 36 37 39 45 46^), both total national expenditure and per person costs (n=9^3 32 35 38 40–44^), per episode of care,^30^ or change in net present value per person and predicted dementia cost value year 2011–2015.^27^

The cost of dementia reported is summarised below, separately as patient level and national level costs, and derivative of absolute cost.

### Costs of dementia per patient

Of the 22 studies^3 23–26 28 29 31–33 35–41 43–46^ which reported cost of dementia per patient, 5 studies^29 31 35 38 42^ provided per capita cost of all-cause dementia, 9 studies^23 24 26 28 32 34 36 37 44^ reported absolute cost of dementia by severity level, 1 study^39^ presented both absolute and attributable costs of dementia by severity, and 1 study^45^ provided the attributable cost of dementia by severity. Two studies^25 33^ estimated per patient cause-specific dementia costs. The WAR studies^3 40 41 43^ estimated per capita costs at the regional level.

The most recent predicted annual total costs of dementia per patient in 2015, in UMICs is US$10 467, in LRMICs is US$3865 and in low-income countries (LICs) is US$939.^3^ Table 2 shows the annual absolute and attributable cost of dementia per patient by severity (n=11 studies), inflated to the year 2019. The total annual absolute per capita cost ranges from US$590.78 (mild) to US$25 510.66 (severe). On average, the indirect cost is the biggest contributor to dementia burden compared with direct cost; however, both cost categories show wide variation. The absolute annual direct cost ranges from US$439.23 (mild) to US$6193.22 (severe). Meanwhile, the indirect cost ranges from US$0 (mild) to US$7428.87 (severe). The total attributable cost ranges from US$0.03 (mild) to US$15.704.82 (severe).

**Table 2:** The annual costs of dementia per patient in LMICs according to cognitive level inflated to year 2019 in US dollar

Study	Country	n	Direct cost (US$)			Indirect cost (US$)			Total cost (US$)		
			Mild	Moderate	Severe	Mild	Moderate	Severe	Mild	Moderate	Severe
Absolute cost											
Aajami *et al*, 2019^23^	Iran	300	590.78	1170.67	2666.67	0	616.64	709.21	590.78	1787.31	3375.88
Allegri *et al,* 2007^24^	Argentina	100	439.23	588.56	1240.19	238.88	263.27	203.44	678.24	851.82	1443.63
Ferretti *et al,* 2018^26^	Brazil	156	627.87	743.89	927.15	6827.71	13 276.62	7428.87	12 095.44	20 369.70	16 468.05
Kongpakwattana *et al*, 2019^28^	Thailand	148	3872.72	5677.09	6193.22	2272.90	3002.96	4777.47	6145.62	8680.04	10 970.68
Koris, 2018^44^	Malaysia	404	NA	NA	NA	NA	NA	NA	3347.95	3884.66	4971.63
Prada *et al,* 2017^39^*	Colombia	340	1151.41	1776.17	5723.13	NA	NA	NA	NA	NA	NA
Rao and Bharath, 2013^32^	India	NA	383.06	1428.89	2286.50	461.21	807.11	1153.02	844.27	2236.00	3439.53
Wang *et al,* 2008^46^	China	66	1680.55	1683.64	1902.71	958.05	1920.83	3755.19	2638.80	3604.68	5658.11
Yan *et al,* 2019^36^	China	3046	NA	NA	NA	NA	NA	NA	13 340.58	16 472.38	25 510.66
Zencir *et al,* 2005^37^*	Turkey	42	1792.27	2626.46	2645.26	160.42	1624.11	2743.73	1953.80	4250.57	5454.27
Mean			1317.24	1961.92	2948.11	1559.88	3073.08	2967.27	4626.17	6904.13	8588.05
Median			889.64	1556.26	2465.88	461.21	1624.11	2743.73	2638.80	3884.66	5454.27
Attributable cost											
Liu, 2013^45^	Cuba	323	97.02	1077.21	432.39	447.46	9934.47	13 574.40	557.44	11 011.68	14 006.79
Liu, 2013^45^	Dominican Republic	242	18.41	445.40	823.73	127.57	3791.62	6460.09	145.98	4236.59	7283.82
Liu, 2013^45^	Peru	166	153.95	1521.06	1363.77	703.92	8448.67	11 668.73	857.86	9970.29	13 033.06
Liu, 2013^45^	Venezuela	145	0.01	0.15	NA	0.02	0.66	NA	0.03	0.81	NA
Liu, 2013^45^	Mexico	180	33.06	143.25	1007.72	254.00	5705.83	14 697.09	287.05	5849.09	15 704.82
Liu, 2013^45^	China	140	35.27	1713.32	2112.41	429.41	8296.82	11 502.56	464.06	10 010.76	13 614.98
Liu, 2013^45^	India	183	14.32	44.59	126.00	114.14	3523.12	5231.09	128.46	3478.94	5357.09
Prada *et al,* 2017^39^*	Colombia	1020	Ref.	171.42	2201.03	NA	NA	NA	NA	NA	NA
Mean attributable cost			50.29	628.40	1152.44	296.64	5671.60	10 522.33	348.70	6365.45	11 500.09
Median attributable cost			33.06	308.41	1007.72	254.00	5705.83	11 585.65	287.05	5849.09	13 324.02

*Direct cost comprises direct medical cost only (no direct non-medical cost).

LMICs, low- and middle-income countries; NA, not available.

All studies except one^26^ showed the costs increase with disease severity, as shown in table 2. We did not extrapolate the cost of patient-level data to national estimates due to a high risk of overestimation or underestimation due to non-representativeness and the small size of samples.

Eleven studies^3 25 29 31 33 35 38 40–43^ with costs of dementia per patient were not included in table 2. Two studies^25 33^ that reported the cost per patient by three common types of dementia showed conflicting results. Frontotemporal dementia (FTD) patients had the highest cost compared with AD and vascular dementia (VaD) in Peru^25^; however, in Argentina,^33^ VaDpatients had the highest annual direct cost compared with AD and FTD. There was significant variation in total costs of dementia of the same country as per the results of five studies^29 31 35 38 42^ from China. The total annual costs of dementia in China as inflated to the year 2019 ranged from US$1332.95^31^ to US$52 163.37^42^ per patient, with median costs of US$7458.36.

### Total national expenditure on dementia patients

Nine out of 24 studies^3 32 35 38 40–44^ reported the total national expenditure or world regional classifications of dementia costs. We calculated the annual cost of dementia as the proportion of the country’s GDP for each country inflated to 2019 as shown in online supplemental table 6. We excluded four studies due to the lack of country-specific estimates^3 40 41^ and the availability of the most recent study from China.^35^ The estimated annual total national costs of dementia ranged from US$1.04 million in Vanuatu to US$195 billion in China. The highest total cost in percentage of GDP was 4.114% in Liberia, and the lowest was 0.001% in Venezuela and Western Sahara.

Table 3 summarises the dementia cost as percentage of GDP for all LMIC groups. The mean total cost of dementia as a proportion of GDP in LMICs was 0.45%. On average, the indirect cost was about 58% of the total costs of dementia. The total costs of dementia as percentage of country’s GDP ranged from 0.35% in LRMICs to 0.46% in LICs. LICs had the lowest direct (0.15%) and the highest indirect cost (0.44%) of dementia. LICs also had the highest total costs percentage of GDP (0.46%), followed by UMICs (0.43%) and LRMICs (0.35%). The most updated total cost of dementia reported in LMICs was US$148.2 billion in 2017, with the highest cost coming from UMICs.^3^ The total LMIC cost inflated to 2019 was US$264.8 billion.

**Table 3:** The national-level direct, indirect and total costs of dementia summarised using the mean estimate percentage of GDP inflated to the year 2019, based on World Bank’s LMICs classification

	Direct cost		Indirect cost		Total cost	
	% of GDP, unweighted	% of GDP, weighted by population	% of GDP, unweighted	% of GDP, weighted by population	% of GDP, unweighted	% of GDP, weighted by population
UMICs	0.24	0.46	0.19	0.45	0.43	0.91
LRMICs	0.17	0.17	0.22	0.17	0.35	0.34
LICs	0.15	0.13	0.44	0.26	0.46	0.36
LMICs	0.19	0.28	0.26	0.30	0.45	0.58

GDP, gross domestic product; LICs, low-income countries; LMICs, low-income and middle-income countries; LRMICs, lower-middle-income countries; UMICs, upper middle-income countries.

### Other dementia costs

Two studies have reported the extent of catastrophic health expenditure caused by dementia. A study in Peru^25^ outlined that the monthly cost of a dementia patient is 2.5 times greater than the current legal minimum wage. Meanwhile, it was found only 0.5% of dementia patients in Malaysia faced an out-of-pocket cost exceeding the 40% threshold for health expenses due to almost total subsidisation of government healthcare.^44^

Only one study^30^ reported the mean direct medical cost of dementia per episode of care, which was MYR10 034 (SD: MYR7604) (approximately US$2414 (SD: US$1830) in year 2017. Severe cases had the highest direct medical cost per episode of care compared with moderate and mild dementia. Meanwhile, one study^27^ showed the national level cost of dementia as the average change in the net present value of GDP of China (US$253.40 per patient in 2015), and the annual predicted value of dementia costs exceeds US$1 trillion by 2050.

### Direct vs indirect costs of dementia

Twenty studies^3 23–29 31 32 35–38 40 42–46^ measured both direct and indirect costs of dementia, and 13 studies^3 26 27 31 32 35–38 40 42 45 46^ showed the driver of total costs is indirect cost ranging from 42% (direct non-medical cost had the same share)^40^ to 94%^35^ of the total costs. Nationally represented data in table 3 also shows that indirect cost is commonly greater than the direct cost in LMICs.

### Determinants of dementia costs

Various factors were identified as the determinants of dementia costs in LMICs. Determinants of greater costs across studies that collected patient-level data are higher dementia severity; n=6^30 32 33 36 39 46^, greater number of comorbidities (n=4^36 38 39 44^), prescription medication (n=3^26 28 29^), longer treatment and hospitalisation (n=2^30 38^), older age (n=1^30^), having a paid caregiver (n=3^23 24 29^), an older informal caregiver (n=1^25^), and lower education level (n=1^26^). Studies using national dementia prevalence or nationally representative data also highlighted greater prevalence of dementia (n=3^3 35 38^), increase in population ageing (n=1^35^) and hospital location outside the capital state (n=1^30^) as the determining factors of higher dementia costs at the national level.

## Discussion

This is the first study to systematically review studies of dementia costs in LMICs, providing a comprehensive understanding of its economic burden. We estimated that the total costs of dementia in LMICs as percentage of GDP is 0.45% in 2019, as compared with 1.4% in HIC in 2017.^3^ The patient-level data showed the annual total costs could be as little as US$590.78 (for mild dementia) to about US$25 510.66 (for severe dementia) per patient. This wide variation of economic burden may be due to the varied number of samples and cost components assessed. Indeed, many of the included studies included patients with greater access to healthcare, with moderately measured indirect costs and assumptions of data sources.

The estimated costs of dementia in LMICs are lower than HIC. The lower cost of dementia in LMICs compared with HICs could be partly due to the differences in the social determinants of health, like the poorer access to clinical care and dementia medications, and lack of knowledge of these services. Most LMICs are under- resourced to provide specialist and hospitalisation services to dementia patients.^47^ Family caregivers offer most of the support even for advanced dementia patients.^48^ These factors could contribute to the lower total cost and a higher proportion of indirect cost in LMICs. However, we cannot prove this as the available data from the articles in the review do not provide any direct evidence to draw this conclusion.

The annual cost of dementia among different countries at the patient level varied widely. The wide range of estimates may be due to the differences in the cost prices, number of samples and specific items being measured in the cost calculation. Although China has multiple economic burden of dementia studies, the costs reported widely varied across studies. This may be due to the inclusion and exclusion of cost components and the method of estimating indirect and direct costs.

Only two studies have assessed the attributable cost of dementia, due to the absence of control sample cost. Even so, one study^39^ managed to include additional expense of dementia associated with the progression of disease using multivariate regression analyses with mild patients as the reference category (no controls in this study). It is difficult to differentiate the real attributable cost of dementia from other health condition.^49 50^ Comparing cost between cohorts with and without dementia is the best alternative to measure attributable cost.^50^

We re-estimated the burden of dementia as a percentage of GDP in 2019 using previous studies reports and found our estimates are similar to the average total costs of dementia as a percentage of GDP in LMICs reported in the most recent WAR 2017^3^ (ie, 0.36% of GDP in LMICs). However, this is expected because only a handful of studies in LMICs provide nationally representative cost data, and our reported figure is heavily based on the previous year WAR estimates. LMICs spend less on dementia compared with the major non-communicable diseases, namely cancer, diabetes, respiratory and cardiovascular diseases which are estimated to be about 4% of the GDP.^51^

The main determinants of dementia costs in LMICs were slightly different from the HICs. Patient care setting, dementia severity and included costs categories and components are some factors outlined by studies from HICs.^52–56^ We also found that the most important factor is dementia severity. Stratification of costs based on dementia severity is important and has been shown as the driver of dementia cost in previous studies.^57 58^ Besides, informal care cost increase 18% per year as dementia progress.^59^ Our finding is also consistent with the previous studies, which employed various diagnosing tools and slightly different cut-off points.^57 58^ Comparison between studies could be better if homogeneous cut-off points and dementia severity diagnosis tool were used. The second most mentioned factor is the number of comorbidities. Cost increases as the number of comorbidities increases. Studies in HICs show that dementia patients with comorbidities are more likely to be hospitalised, have longer hospital stays, and spend more on comorbidities than people without dementia.^3 60–62^

The cost of dementia studies in this review largely represents the UMICs group. Lack of nationally representative data from LMICs is a concern, despite most studies having minimal risk of bias and detailed analyses. Most of these studies use convenient sampling or non-random participant recruitment from a single healthcare centre or dementia society. These patients tend to have advanced dementia, their caregivers typically report higher strain levels, and the families have greater access and use more healthcare and community support services.^40^ Limited financial and human capacity is the most common barrier that lead to challenges in conducting clinical studies in developing countries.^63^ In LMICs, convenient sampling may cause significant bias in the cost estimation of dementia, as patients have inequitable access to healthcare.^59^ Therefore, the results shown may have low generalisability to broader dementia patients within the same country.

Indirect costs are often challenging to be estimated. One of the reasons is dementia is prevalent in older adults and mostly retirees; therefore, productivity loss (loss of income due to unpaid leave/absenteeism, early retirement, or death) of the patient is less often described in the cost of illness studies on dementia. Our review found that only a few studies measured productivity loss of a caregiver but with no justification for the exclusion of patient’s productivity loss. Indirect costing was based only on informal caregiving time, which may have underestimated the magnitude of caregivers’ and patients' loss of resources. It is important that future studies justify their decision in the methodology if productivity loss of either patient or caregiver is excluded.

The indirect cost due to the poorer health of caregivers and lost wages associated with caregiving are often under-reported in these studies. Provision for the training of professional and family carers is a key strategy to reduce the negative outcomes associated with caregiving.^64 65^ However, the biggest challenge would be to mobilise resources to cater to a large number of carers with the limited resources available. In this context, digital health interventions to provide distant training, many of which have been proven effective in the COVID-19 pandemic, can be adapted to increase the reach of carer training in dementia.^66^

The impact of caregiving on informal caregivers’ health and its associated cost is often overlooked.^60 62^ Thus, data of indirect cost from health deterioration due to caregiving even from HICs are unavailable for cost modelling.^40^ A hidden cost component associated with dementia caregiving is the long-term cost of deteriorating health and the resulting impact on the health and productivity of the carers. There should be standardised ways of capturing this cost in dementia costing studies. Caregiving time often been highlighted as the major contributor to the cost of dementia, regardless of the economic capacity of the country.^3^ In HICs such as the USA, a combination of informal care and paid formal care had the highest contribution to total dementia costs.^61^ In LMICs, indirect cost from informal caregiving is the major contributor to the total cost burden of dementia compared with direct cost. Better estimation and inclusion of indirect cost parameters may increase the actual dementia costs estimate in LMICs.

The strength of this study is that this is the first systematic review of dementia costs from LMICs regardless of the publication year and language. The methodological aspect of the included studies was also evaluated, providing recommendations for future research studies. There are some limitations to our study. Although we wanted to include as many studies as possible from LMICs, only 15 countries had original published costs data that were included in the review. We inflated the cost of dementia at the national level to the year 2019 and showed them as a percentage of GDP without accounting for current dementia prevalence, as there is limited data on the current prevalence of dementia in each country. This may have resulted in an underestimation of cost. All included studies had participants who were at least 60 years old, implying a lack of data on costs associated with early-onset dementias in LMICs. Lastly, most studies used data from UMICs. This may limit the generalisability of the summarised data.

## Conclusion

Dementia is associated with a significant cost in LMICs. However, is difficult to draw accurate conclusions on the actual economic burden due to the lack of available data and standardised cost assessment methods. Future studies on dementia costs needed to be standardised and inclusive of all important cost items. The stakeholder in LMICs should focus on the delivery of holistic primary healthcare to slowdown dementia progression and prevention of comorbidities, that may reduce the direct cost of dementia in the long run. Besides, LMICs should enhance the assistance offered to caregivers of people living with dementia to reduce the burden of caregiving and thereby the indirect cost.

## Data Availability

Data are available on reasonable request.

## References

[1] Guerchet M, Prince M, et al, Alzheimer’s Disease International. Numbers of people with dementia around the world 2020.

[2] Prince MJ. World Alzheimer report 2015: the global impact of dementia: an analysis of prevalence, incidence, cost and trends. Alzheimer’s Disease International, 2015.

[3] Wimo A, Guerchet M, Ali G-C, et al. The worldwide costs of dementia 2015 and comparisons with 2010. Alzheimers Dement 2017;13:1–7. 10.1016/j.jalz.2016.07.150 27583652 PMC5232417

[4] Agüero-Torres H, Fratiglioni L, Winblad B. Natural history of Alzheimer’s disease and other dementias: review of the literature in the light of the findings from the Kungsholmen project. Int J Geriatr Psychiatry 1998;13:755–66. 10.1002/(sici)1099-1166(1998110)13:11&lt;755::aid-gps862&gt;3.0.co;2-y 9850872

[5] Leung GM, Yeung RYT, Chi I, et al. The economics of Alzheimer disease. Dement Geriatr Cogn Disord 2003;15:34–43. 10.1159/000066675 12457077

[6] Quentin W, Riedel-Heller SG, Luppa M, et al. Cost-of-illness studies of dementia: a systematic review focusing on stage dependency of costs. Acta Psychiatr Scand 2010;121:243–59. 10.1111/j.1600-0447.2009.01461.x 19694634

[7] Schaller S, Mauskopf J, Kriza C, et al. The main cost drivers in dementia: a systematic review. Int J Geriatr Psychiatry 2015;30:111–29. 10.1002/gps.4198 25320002

[8] Wimo A, Ljunggren G, Winblad B. Costs of dementia and dementia care: a review. Int J Geriatr Psychiatry 1997;12:841–56. 10.1002/(SICI)1099-1166(199708)12:8<841::AID-GPS652>3.0.CO;2-R 9283930

[9] Hojman DA, Duarte F, Ruiz-Tagle J, et al. The cost of dementia in an unequal country: the case of Chile. PLoS One 2017;12:e0172204. 10.1371/journal.pone.0172204 28267795 PMC5340351

[10] Jutkowitz E, Kuntz KM, Dowd B, et al. Effects of cognition, function, and behavioral and psychological symptoms on out-of-pocket medical and nursing home expenditures and time spent caregiving for persons with dementia. Alzheimers Dement 2017;13:801–9. 10.1016/j.jalz.2016.12.011 28161279 PMC5644025

[11] Wimo A, Jönsson L, Fratiglioni L, et al. The societal costs of dementia in Sweden 2012 - relevance and methodological challenges in valuing informal care. Alzheimers Res Ther 2016;8:59. 10.1186/s13195-016-0215-9 27986093 PMC5162098

[12] Hopkins S. Health expenditure comparisons: low, middle and high income countries. Open Health Services Policy J 2010;3:21–7.

[13] Kalaria RN, Maestre GE, Arizaga R, et al. Alzheimer’s disease and vascular dementia in developing countries: prevalence, management, and risk factors. Lancet Neurol 2008;7:812–26. 10.1016/S1474-4422(08)70169-8 18667359 PMC2860610

[14] Page MJ, McKenzie JE, Bossuyt PM, et al. The PRISMA 2020 statement: an updated guideline for reporting systematic reviews. BMJ 2021;372:n71. 10.1136/bmj.n71 33782057 PMC8005924

[15] The World Bank. World bank country and lending groups, 2020. Available: https://datahelpdesk.worldbank.org/knowledgebase/articles/906519-world-bank-country-and-lending-groups [Accessed 12 May 2020].

[16] Larg A, Moss JR. Cost-of-illness studies. Pharmacoeconomics 2011;29:653–71. 10.2165/11588380-000000000-00000 21604822

[17] The World Bank. Consumer price index (2010 = 100), 2020. Available: https://data.worldbank.org/indicator/FP.CPI.TOTL [Accessed 12 May 2020].

[18] Turner HC, Lauer JA, Tran BX, et al. Adjusting for inflation and currency changes within health economic studies. Value Health 2019;22:1026–32. 10.1016/j.jval.2019.03.021 31511179

[19] The world Bank. World bank open data, 2020. Available: https://data.worldbank.org/ [Accessed 12 May 2020].

[20] International Monetary Fund, 2020. Available: https://data.imf.org/?sk=388DFA60-1D26-4ADE-B505-A05A558D9A42&sId=1479329334655

[21] Thaver A, Ahmad A. Economic perspective of dementia care in Pakistan. Neurology 2018;90:e993–4. 10.1212/WNL.0000000000005108 29530970

[22] Uwakwe R. The financial (material) consequences of dementia care in a developing country: Nigeria. Alzheimer Dis Assoc Disord 2001;15:56–7. 10.1097/00002093-200101000-00008 11236826

[23] Aajami Z, Kebriaeezadeh A, Nikfar S. Direct and indirect cost of managing Alzheimer’s disease in the Islamic Republic of Iran. Iran J Neurol 2019;18:7–12. 31316730 PMC6626604

[24] Allegri RF, Butman J, Arizaga RL, et al. Economic impact of dementia in developing countries: an evaluation of costs of Alzheimer-type dementia in Argentina. Int Psychogeriatr 2007;19:705–18. 10.1017/S1041610206003784 16870037

[25] Custodio N, Lira D, Herrera-Perez E, et al. Cost-of-illness study in a retrospective cohort of patients with dementia in Lima, Peru. Dement Neuropsychol 2015;9:32–41. 10.1590/S1980-57642015DN91000006 29213939 PMC5618989

[26] Ferretti C, Sarti FM, Nitrini R, et al. An assessment of direct and indirect costs of dementia in Brazil. PLoS One 2018;13:e0193209. 10.1371/journal.pone.0193209 29494693 PMC5832239

[27] Keogh-Brown MR, Jensen HT, Arrighi HM, et al. The impact of Alzheimer’s disease on the Chinese economy. EBioMedicine 2016;4:184–90. 10.1016/j.ebiom.2015.12.019 26981556 PMC4776062

[28] Kongpakwattana K, Dejthevaporn C, Krairit O, et al. A real-world evidence analysis of associations among costs, quality of life, and disease-severity indicators of Alzheimer’s disease in Thailand. Value Health 2019;22:1137–45. 10.1016/j.jval.2019.04.1937 31563256

[29] Mould-Quevedo JF, Tang B, Harary E, et al. The burden of caring for dementia patients: caregiver reports from a cross-sectional hospital-based study in China. Expert Rev Pharmacoecon Outcomes Res 2013;13:663–73. 10.1586/14737167.2013.838029 24138651

[30] Nur AM, Aljunid SM, Ismail N. Provider costs of treating dementia among the elderly in government hospitals of Malaysia. Malaysia J Public Health Med 2017:121–7.

[31] Prince M, Quraishi S, Copeland J, 10/66 Dementia Research Group. Care arrangements for people with dementia in developing countries. Int J Geriatr Psychiatry 2004;19:170–7. 10.1002/gps.1046 14758582

[32] Rao GN, Bharath S. Cost of dementia care in India: delusion or reality? Indian J Public Health 2013;57:71–7. 10.4103/0019-557X.114986 23873192

[33] Rojas G, Bartoloni L, Dillon C, et al. Clinical and economic characteristics associated with direct costs of Alzheimer’s, frontotemporal and vascular dementia in Argentina. Int Psychogeriatr 2011;23:554–61. 10.1017/S1041610210002012 21044400

[34] Wang L, Si L, Cocker F, et al. A systematic review of cost-of-illness studies of multimorbidity. Appl Health Econ Health Policy 2018;16:15–29. 10.1007/s40258-017-0346-6 28856585

[35] Xu J, Wang J, Wimo A, et al. The economic burden of dementia in China, 1990-2030: implications for health policy. Bull World Health Organ 2017;95:18–26. 10.2471/BLT.15.167726 28053361 PMC5180346

[36] Yan X, Li F, Chen S, et al. Associated factors of total costs of Alzheimer’s disease: a cluster-randomised observational study in China. J Alzheimer’s Dis 2019;69:795–806. 10.3233/JAD-190166 31156170

[37] Zencir M, Kuzu N, Beşer NG, et al. Cost of Alzheimer’s disease in a developing country setting. Int J Geriatr Psychiatry 2005;20:616–22. 10.1002/gps.1332 16021668

[38] Jia J, Wei C, Chen S, et al. The cost of Alzheimer’s disease in China and re‐estimation of costs worldwide. Alzheimer's & Dementia 2018;14:483–91. 10.1016/j.jalz.2017.12.006 29433981

[39] Prada SI, Takeuchi Y, Merchán-Galvis AM, et al. Actual expense associated with patients with Alzheimer’s disease in Colombia. Int Psychogeriatr 2017;29:1835–40. 10.1017/S1041610217000710 28592351

[40] Wimo A, Jönsson L, Bond J, et al. The worldwide economic impact of dementia 2010. Alzheimers Dement 2013;9:1–11. 10.1016/j.jalz.2012.11.006 23305821

[41] Wimo A, Jonsson L, Winblad B. An estimate of the worldwide prevalence and direct costs of dementia in 2003. Dement Geriatr Cogn Disord 2006;21:175–81. 10.1159/000090733 16401889

[42] Wimo A, Winblad B, Jönsson L. An estimate of the total worldwide societal costs of dementia in 2005. Alzheimers Dement 2007;3:81–91. 10.1016/j.jalz.2007.02.001 19595921

[43] Wimo A, Winblad B, Jönsson L. The worldwide societal costs of dementia: estimates for 2009. Alzheimers Dement 2010;6:98–103. 10.1016/j.jalz.2010.01.010 20298969

[44] Koris RB. Economic burden of dementia and healthcare costs of demented Elerly in Malaysia. Universiti Putra Malaysia, 2018.

[45] Liu Z. Economic costs of dementia in low and middle income countries. King’s College London, 2013.

[46] Wang G, Cheng Q, Zhang S, et al. Economic impact of dementia in developing countries: an evaluation of Alzheimer-type dementia in Shanghai, China. J Alzheimers Dis 2008;15:109–15. 10.3233/jad-2008-15109 18780971

[47] Prince M, Comas-Herrera A, Knapp M. World Alzheimer report 2016: improving healthcare for people living with dementia: coverage, quality and costs now and in the future 2016.

[48] Fam J, Mahendran R, Kua EH. Dementia care in low and middle-income countries. Curr Opin Psychiatry 2019;32:461–4. 10.1097/YCO.0000000000000523 31082844

[49] Wimo A, Prince M. The global economic impact of dementia. World Alzheimer Report 2010;2010:12.

[50] Bloom BS, de Pouvourville N, Straus WL. Cost of illness of Alzheimer’s disease: how useful are current estimates? Gerontologist 2003;43:158–64. 10.1093/geront/43.2.158 12677073

[51] World Health Organization and World Economic Forum. From burden to ‘best buys’: reducing the economic impact of non-communicable diseases in low and middle-income countries. Geneva: World Economic Forum, 2011.

[52] Costa N, Derumeaux H, Rapp T, et al. Methodological considerations in cost of illness studies on Alzheimer disease. Health Econ Rev 2012;2:18. 10.1186/2191-1991-2-18 22963680 PMC3563616

[53] Gustavsson A, Jonsson L, Rapp T, et al. Differences in resource use and costs of dementia care between European countries: baseline data from the ICTUS study. J Nutr Health Aging 2010;14:648–54. 10.1007/s12603-010-0311-720922341

[54] Jönsson L, Wimo A. The cost of dementia in Europe: a review of the evidence, and methodological considerations. Pharmacoeconomics 2009;27:391–403. 10.2165/00019053-200927050-00004 19586077

[55] Mauskopf J, Mucha L. A Review of the Methods Used to Estimate the Cost of Alzheimer’s Disease in the United States. Am J Alzheimers Dis Other Demen 2011;26:298–309. 10.1177/1533317511407481 21561991 PMC10845619

[56] Mauskopf J, Racketa J, Sherrill E. Alzheimer’s disease: the strength of association of costs with different measures of disease severity. J Nutr Health Aging 2010;14:655–63. 10.1007/s12603-010-0312-620922342

[57] Bynum JPW, Rabins PV, Weller W, et al. The relationship between a dementia diagnosis, chronic illness, Medicare expenditures, and hospital use. J Am Geriatr Soc 2004;52:187–94. 10.1111/j.1532-5415.2004.52054.x 14728626

[58] Rattinger GB, Schwartz S, Mullins CD, et al. Dementia severity and the longitudinal costs of informal care in the Cache County population. Alzheimers Dement 2015;11:946–54. 10.1016/j.jalz.2014.11.004 25614127 PMC4506892

[59] Maestre GE. Assessing dementia in resource-poor regions. Curr Neurol Neurosci Rep 2012;12:511–9. 10.1007/s11910-012-0300-9 22864986 PMC3434885

[60] Deb A, Thornton JD, Sambamoorthi U, et al. Direct and indirect cost of managing Alzheimer's disease and related dementias in the United States. Expert Rev Pharmacoecon Outcomes Res 2017;17:189–202. 10.1080/14737167.2017.1313118 28351177 PMC5494694

[61] Hurd MD, Martorell P, Delavande A, et al. Monetary costs of dementia in the United States. N Engl J Med 2013;368:1326–34. 10.1056/NEJMsa1204629 23550670 PMC3959992

[62] Rahman A, Anjum R, Sahakian Y. Impact of caregiving for dementia patients on healthcare utilization of caregivers. Pharmacy 2019;7:138. 10.3390/pharmacy7040138 PMC6958358 31554156

[63] Alemayehu C, Mitchell G, Nikles J. Barriers for conducting clinical trials in developing countries- a systematic review. Int J Equity Health 2018;17:37. 10.1186/s12939-018-0748-6 29566721 PMC5863824

[64] Birkenhäger-Gillesse EG, Achterberg WP, Janus SIM, et al. Effects of caregiver dementia training in caregiver-patient dyads: a randomized controlled study. Int J Geriatr Psychiatry 2020;35:1376–84. 10.1002/gps.5378 32662184 PMC7689696

[65] Brodaty H, McGilchrist C, Harris L, et al. Time until institutionalization and death in patients with dementia. Role of caregiver training and risk factors. Arch Neurol 1993;50:643–50. 10.1001/archneur.1993.00540060073021 8503802

[66] Egan KJ, Pinto-Bruno Ángel C, Bighelli I, et al. Online training and support programs designed to improve mental health and reduce burden among caregivers of people with dementia: a systematic review. J Am Med Dir Assoc 2018;19:200–6. 10.1016/j.jamda.2017.10.023 29306605

