# Supplementary material for: The economic burden of dementia in low- and middle-income countries (LMICs): a systematic review
Source: BMJ Glob Health. 2022 Apr 3;7(4):e007409. doi: 10.1136/bmjgh-2021-007409 (PMC8981345; doi:10.1136/bmjgh-2021-007409)
Supplement: Supplementary data [file bmjgh-2021-007409supp001.pdf]

Economic burden of dementia in low- and middle- income countries (LMICs): A systematic review.

Appendix

Search strategy

MEDLINE/PUBMED

Filter: Humans

Keywords

Dementia or Alzheimer[1]

((("dementia"[MeSH Terms] OR "dementia"[All Fields]) OR "dementias"[All Fields]) OR "dementia s"[All Fields]) OR ((((((("alzheime s"[All Fields] OR "alzheimer disease"[MeSH Terms]) OR ("alzheimer"[All Fields] AND "disease"[All Fields])) OR "alzheimer disease"[All Fields]) OR "alzheimer"[All Fields]) OR "alzheimers"[All Fields]) OR "alzheimer s"[All Fields]) OR "alzheimers s"[All Fields])

AND

Cost-of-illness[2]

"forecasting"[MeSH Terms] OR "health expenditures"[MeSH Terms] OR "spending"[Title/Abstract] OR "costs and cost analysis"[MeSH Terms] OR "cost-of-illness"[Title/Abstract] OR "cost-of-illness"[Title/Abstract]

[1]We follow the keywords used in Killin et al. (2016), Environmental risk factors for dementia: a systematic review. *BMC Geriatrics*, 16(1), 175.

[2]We follow the keywords used in Wang et al. (2018), A Systematic Review of Cost-of-Illness Studies of Multimorbidity. Appl Health Econ Health Policy, 16(1), 15-29.

AND

Low- and middle- income countries (LMICs)

Using EPOC LMIC filter 2020_v3 #1a OR #2 https://epoc.cochrane.org/lmic-filters

#1a

afghanistan[MeSH] OR albania[MeSH] OR algeria[MeSH] OR american samoa[MeSH] OR angola[MeSH] OR antigua and barbuda[MeSH] OR argentina[MeSH] OR armenia[MeSH] OR aruba[MeSH] OR azerbaijan[MeSH] OR bahrain[MeSH] OR bangladesh[MeSH] OR

barbados[MeSH] OR republic of belarus[MeSH] OR belize[MeSH] OR benin[MeSH] OR bhutan[MeSH] OR bolivia[MeSH] OR bosnia and herzegovina[MeSH] OR botswana[MeSH] OR brazil[MeSH] OR bulgaria[MeSH] OR burkina faso[MeSH] OR burundi[MeSH] OR cabo verde[MeSH] OR cambodia[MeSH] OR cameroon[MeSH] OR central african republic[MeSH] OR chad[MeSH] OR chile[MeSH] OR china[MeSH] OR colombia[MeSH] OR comoros[MeSH] OR democratic republic of the congo[MeSH] OR congo[MeSH] OR costa rica[MeSH] OR cote d'ivoire[MeSH] OR croatia[MeSH] OR cuba[MeSH] OR cyprus[MeSH] OR czech republic[MeSH] OR djibouti[MeSH] OR dominica[MeSH] OR dominican republic[MeSH] OR ecuador[MeSH] OR egypt[MeSH] OR el salvador[MeSH] OR equatorial guinea[MeSH] OR eritrea[MeSH] OR estonia[MeSH] OR swaziland[MeSH] OR ethiopia[MeSH] OR fiji[MeSH] OR gabon[MeSH] OR gambia[MeSH] OR georgia (republic)[MeSH] OR ghana[MeSH] OR gibraltar[MeSH] OR greece[MeSH] OR grenada[MeSH] OR guam[MeSH] OR guatemala[MeSH] OR guinea[MeSH] OR guinea bissau[MeSH] OR guyana[MeSH] OR haiti[MeSH] OR honduras[MeSH] OR hungary[MeSH] OR india[MeSH] OR indonesia[MeSH] OR iran[MeSH] OR iraq[MeSH] OR jamaica[MeSH] OR jordan[MeSH] OR kazakhstan[MeSH] OR kenya[MeSH] OR democratic people's republic of korea[MeSH] OR republic of korea[MeSH] OR kosovo[MeSH] OR kyrgyzstan[MeSH] OR laos[MeSH] OR latvia[MeSH] OR lebanon[MeSH] OR lesotho[MeSH] OR liberia[MeSH] OR libya[MeSH] OR lithuania[MeSH] OR macau[MeSH] OR macedonia (republic)[MeSH] OR madagascar[MeSH] OR malawi[MeSH] OR malaysia[MeSH] OR indian ocean islands[MeSH] OR mali[MeSH] OR malta[MeSH] OR micronesia[MeSH] OR palau[MeSH] OR mauritania[MeSH] OR mauritius[MeSH] OR mexico[MeSH] OR moldova[MeSH] OR mongolia[MeSH] OR montenegro[MeSH] OR morocco[MeSH] OR mozambique[MeSH] OR myanmar[MeSH] OR namibia[MeSH] OR nepal[MeSH] OR netherlands antilles[MeSH] OR nicaragua[MeSH] OR niger[MeSH] OR nigeria[MeSH] OR oman[MeSH] OR pakistan[MeSH] OR panama[MeSH] OR papua new guinea[MeSH] OR paraguay[MeSH] OR peru[MeSH] OR philippines[MeSH] OR poland[MeSH] OR portugal[MeSH] OR puerto rico[MeSH] OR romania[MeSH] OR russia[MeSH] OR rwanda[MeSH] OR samoa[MeSH] OR sao tome and principe[MeSH] OR saudi arabia[MeSH] OR senegal[MeSH] OR serbia[MeSH] OR seychelles[MeSH] OR sierra leone[MeSH] OR slovakia[MeSH] OR slovenia[MeSH] OR melanesia[MeSH] OR somalia[MeSH] OR south africa[MeSH] OR south sudan[MeSH] OR sri lanka[MeSH] OR saint kitts and nevis[MeSH] OR saint lucia[MeSH] OR saint vincent and the grenadines[MeSH] OR sudan[MeSH] OR suriname[MeSH] OR syria[MeSH] OR tajikistan[MeSH] OR tanzania[MeSH] OR thailand[MeSH] OR timor leste[MeSH] OR togo[MeSH] OR tonga[MeSH] OR trinidad and tobago[MeSH] OR tunisia[MeSH] OR turkey (republic)[MeSH] OR turkmenistan[MeSH] OR uganda[MeSH] OR ukraine[MeSH] OR uruguay[MeSH] OR uzbekistan[MeSH] OR vanuatu[MeSH] OR venezuela[MeSH] OR vietnam[MeSH] OR middle east[MeSH] OR yemen[MeSH] OR yugoslavia[MeSH] OR zambia[MeSH] OR zimbabwe[MeSH] OR africa south of the sahara[MeSH] OR africa, central[MeSH] OR africa, northern[MeSH] OR africa, southern[MeSH] OR africa, eastern[MeSH] OR africa, western[MeSH] OR west indies[MeSH] OR indian ocean islands[MeSH] OR caribbean region[MeSH] OR central america[MeSH] OR latin america[MeSH] OR south america[MeSH] OR asia, central[MeSH] OR asia, northern[MeSH] OR asia, southeastern[MeSH] OR asia, western[MeSH] OR europe, eastern[MeSH] OR developing countries[MeSH]

OR

#2

afghanistan**[Text Word]** OR albania[Text Word] OR algeria[Text Word] OR american samoa[Text Word] OR angola[Text Word] OR antigua[Text Word] OR barbuda[Text Word] OR argentina[Text Word] OR armenia[Text Word] OR armenian[Text Word] OR aruba[Text Word] OR azerbaijan[Text Word] OR bahrain[Text Word] OR bangladesh[Text Word] OR barbados[Text Word] OR belarus[Text Word] OR byelarus[Text Word] OR belorussia[Text Word] OR byelorussian[Text Word] OR belize[Text Word] OR british honduras[Text Word] OR benin[Text Word] OR dahomey[Text Word] OR bhutan[Text Word] OR bolivia[Text Word] OR bosnia[Text Word] OR herzegovina[Text Word] OR botswana[Text Word] OR bechuanaland[Text Word] OR brazil[Text Word] OR brasil[Text Word] OR bulgaria[Text Word] OR burkina faso[Text Word] OR burkina fasso[Text Word] OR upper volta[Text Word] OR burundi[Text Word] OR urundi[Text Word] OR cabo verde[Text Word] OR cape verde[Text Word] OR cambodia[Text Word] OR kampuchea[Text Word] OR khmer republic[Text Word] OR cameroon[Text Word] OR cameron[Text Word] OR cameroun[Text Word] OR central african republic[Text Word] OR ubangi shari[Text Word] OR chad[Text Word] OR chile[Text Word] OR china[Text Word] OR colombia[Text Word] OR comoros[Text Word] OR comoro islands[Text Word] OR mayotte[Text Word] OR congo[Text Word] OR zaire[Text Word] OR costa rica[Text Word] OR cote d'ivoire[Text Word] OR cote d'ivoire[Text Word] OR cote d'ivoire[Text Word] OR ivory coast[Text Word] OR croatia[Text Word] OR cuba[Text Word] OR cyprus[Text Word] OR czech republic[Text Word] OR czechoslovakia[Text Word] OR djibouti[Text Word] OR french somaliland[Text Word] OR dominica[Text Word] OR dominican republic[Text Word] OR ecuador[Text Word] OR egypt[Text Word] OR united arab republic[Text Word] OR el salvador[Text Word] OR equatorial guinea[Text Word] OR spanish guinea[Text Word] OR eritrea[Text Word] OR estonia[Text Word] OR eswatini[Text Word] OR swaziland[Text Word] OR ethiopia[Text Word] OR fiji[Text Word] OR gabon[Text Word] OR gabonese republic[Text Word] OR gambia[Text Word] OR georgia[Text Word] OR georgian[Text Word] OR ghana[Text Word] OR gold coast[Text Word] OR gibraltar[Text Word] OR greece[Text Word] OR grenada[Text Word] OR guam[Text Word] OR guatemala[Text Word] OR guinea[Text Word] OR guyana[Text Word] OR guiana[Text Word] OR haiti[Text Word] OR hispaniola[Text Word] OR honduras[Text Word] OR hungary[Text Word] OR india[Text Word] OR indonesia[Text Word] OR timor[Text Word] OR iran[Text Word] OR iraq[Text Word] OR isle of man[Text Word] OR jamaica[Text Word] OR jordan[Text Word] OR

kazakhstan[Text Word] OR kazakh[Text Word] OR kenya[Text Word] OR korea[Text Word] OR kosovo[Text Word] OR kyrgyzstan[Text Word] OR kirghizia[Text Word] OR kirgizstan[Text Word] OR kyrgyz republic[Text Word] OR kirghiz[Text Word] OR laos[Text Word] OR lao pdr[Text Word] OR lao people's democratic republic[Text Word] OR latvia[Text Word] OR lebanon[Text Word] OR lesotho[Text Word] OR basutoland[Text Word] OR liberia[Text Word] OR libya[Text Word] OR libyan arab jamahiriya[Text Word] OR lithuania[Text Word] OR macau[Text Word] OR macao[Text Word] OR macedonia[Text Word] OR madagascar[Text Word] OR malagasy republic[Text Word] OR malawi[Text Word] OR nyasaland[Text Word] OR malaysia[Text Word] OR maldives[Text Word] OR indian ocean[Text Word] OR mali[Text Word] OR malta[Text Word] OR micronesia[Text Word] OR kiribati[Text Word] OR marshall islands[Text Word] OR nauru[Text Word] OR northern mariana islands[Text Word] OR palau[Text Word] OR tuvalu[Text Word] OR mauritania[Text Word] OR mauritius[Text Word] OR mexico[Text Word] OR moldova[Text Word] OR moldovian[Text Word] OR mongolia[Text Word] OR montenegro[Text Word] OR morocco[Text Word] OR ifni[Text Word] OR mozambique[Text Word] OR portuguese east africa[Text Word] OR myanmar[Text Word] OR burma[Text Word] OR namibia[Text Word] OR nepal[Text Word] OR netherlands antilles[Text Word] OR nicaragua[Text Word] OR niger[Text Word] OR nigeria[Text Word] OR oman[Text Word] OR muscat[Text Word] OR pakistan[Text Word] OR panama[Text Word] OR papua new guinea[Text Word] OR paraguay[Text Word] OR peru[Text Word] OR philippines[Text Word] OR philipines[Text Word] OR phillipines[Text Word] OR phillippines[Text Word] OR poland[Text Word] OR polish people's republic[Text Word] OR portugal[Text Word] OR portuguese republic[Text Word] OR puerto rico[Text Word] OR romania[Text Word] OR russia[Text Word] OR russian federation[Text Word] OR ussr[Text Word] OR soviet union[Text Word] OR union of soviet socialist republics[Text Word] OR rwanda[Text Word] OR ruanda[Text Word] OR samoa[Text Word] OR pacific islands[Text Word] OR polynesia[Text Word] OR samoan islands[Text Word] OR sao tome and principe[Text Word] OR saudi arabia[Text Word] OR senegal[Text Word] OR serbia[Text Word] OR seychelles[Text Word] OR sierra leone[Text Word] OR slovakia[Text Word] OR slovak republic[Text Word] OR slovenia[Text Word] OR melanesia[Text Word] OR solomon island[Text Word] OR solomon islands[Text Word] OR norfolk island[Text Word] OR somalia[Text Word] OR south africa[Text Word] OR south sudan[Text Word] OR sri lanka[Text Word] OR ceylon[Text Word] OR saint kitts and nevis[Text Word] OR st kitts and nevis[Text Word] OR saint lucia[Text Word] OR st lucia[Text Word] OR saint vincent[Text Word] OR st vincent[Text Word] OR grenadines[Text Word] OR sudan[Text Word] OR suriname[Text Word] OR surinam[Text

Word] OR syria[Text Word] OR syrian arab republic[Text Word] OR tajikistan[Text Word] OR tadjikistan[Text Word] OR tadzhikistan[Text Word] OR tadzhik[Text Word] OR tanzania[Text Word] OR tanganyika[Text Word] OR thailand[Text Word] OR siam[Text Word] OR timor leste[Text Word] OR east timor[Text Word] OR togo[Text Word] OR togolese republic[Text Word] OR tonga[Text Word] OR trinidad[Text Word] OR tobago[Text Word] OR tunisia[Text Word] OR turkey[Text Word] OR turkmenistan[Text Word] OR turkmen[Text Word] OR uganda[Text Word] OR ukraine[Text Word] OR uruguay[Text Word] OR uzbekistan[Text Word] OR uzbek[Text Word] OR vanuatu[Text Word] OR new hebrides[Text Word] OR venezuela[Text Word] OR vietnam[Text Word] OR viet nam[Text Word] OR middle east[Text Word] OR west bank[Text Word] OR gaza[Text Word] OR palestine[Text Word] OR yemen[Text Word] OR yugoslavia[Text Word] OR zambia[Text Word] OR zimbabwe[Text Word] OR northern rhodesia[Text Word] OR global south[Text Word] OR africa south of the sahara[Text Word] OR sub saharan africa[Text Word] OR subsaharan africa[Text Word] OR central africa[Text Word] OR north africa[Text Word] OR northern africa[Text Word] OR magreb[Text Word] OR maghrib[Text Word] OR sahara[Text Word] OR southern africa[Text Word] OR east africa[Text Word] OR eastern africa[Text Word] OR west africa[Text Word] OR western africa[Text Word] OR west indies[Text Word] OR indian ocean islands[Text Word] OR caribbean[Text Word] OR central america[Text Word] OR latin america[Text Word] OR south america[Text Word] OR central asia[Text Word] OR north asia[Text Word] OR northern asia[Text Word] OR southeastern asia[Text Word] OR south eastern asia[Text Word] OR southeast asia[Text Word] OR south east asia[Text Word] OR western asia[Text Word] OR east europe[Text Word] OR eastern europe[Text Word] OR developing country[Text Word] OR developing countries[Text Word] OR developing nation[Text Word] OR developing nations[Text Word] OR developing population[Text Word] OR developing populations[Text Word] OR developing world[Text Word] OR less developed country[Text Word] OR less developed countries[Text Word] OR less developed nation[Text Word] OR less developed nations[Text Word] OR less developed world[Text Word] OR lesser developed countries[Text Word] OR lesser developed nations[Text Word] OR under developed country[Text Word] OR under developed countries[Text Word] OR under developed nations[Text Word] OR under developed world[Text Word] OR underdeveloped country[Text Word] OR underdeveloped countries[Text Word] OR underdeveloped nation[Text Word] OR underdeveloped nations[Text Word] OR underdeveloped population[Text Word] OR underdeveloped populations[Text Word] OR underdeveloped world[Text Word] OR middle income country[Text Word] OR middle income countries[Text Word] OR middle income nation[Text

Word] OR middle income nations[Text Word] OR middle income population[Text Word] OR middle income populations[Text Word] OR low income country[Text Word] OR low income countries[Text Word] OR low income nation[Text Word] OR low income nations[Text Word] OR low income population[Text Word] OR low income populations[Text Word] OR lower income country[Text Word] OR lower income countries[Text Word] OR lower income nations[Text Word] OR lower income population[Text Word] OR lower income populations[Text Word] OR underserved countries[Text Word] OR underserved nations[Text Word] OR underserved population[Text Word] OR underserved populations[Text Word] OR under served population[Text Word] OR under served populations[Text Word] OR deprived countries[Text Word] OR deprived population[Text Word] OR deprived populations[Text Word] OR poor country[Text Word] OR poor countries[Text Word] OR poor nation[Text Word] OR poor nations[Text Word] OR poor population[Text Word] OR poor populations[Text Word] OR poor world[Text Word] OR poorer countries[Text Word] OR poorer nations[Text Word] OR poorer population[Text Word] OR poorer populations[Text Word] OR developing economy[Text Word] OR developing economies[Text Word] OR less developed economy[Text Word] OR less developed economies[Text Word] OR underdeveloped economies[Text Word] OR middle income economy[Text Word] OR middle income economies[Text Word] OR low income economy[Text Word] OR low income economies[Text Word] OR lower income economies[Text Word] OR low gdp[Text Word] OR low gnp[Text Word] OR low gross domestic[Text Word] OR low gross national[Text Word] OR lower gdp[Text Word] OR lower gross domestic[Text Word] OR lmic[Text Word] OR lmics[Text Word] OR third world[Text Word] OR lami country[Text Word] OR lami countries[Text Word] OR transitional country[Text Word] OR transitional countries[Text Word] OR emerging economies[Text Word] OR emerging nation[Text Word] OR emerging nations[Text Word]

Mattap SM, *et al. BMJ Global Health* 2022; 7:e007409. doi: 10.1136/bmjgh-2021-007409

Table 1. Risk of bias assessment based on Larg and Moss method.

**Question/Study**	Aajami, 2019	Allegri, 2007	Custodio, 2015	Ferretti, 2018	Jia, 2018	Keogh-Brown, 2015	Kongpak wattana, 2019	Koris, 2018	Liu, 2013	Mould-Quevedo, 2013	Nur, 2017	Prada, 2017
**1) Analytical framework: what costs should have been measured? (overall)**												
(a) What was the motivation												
and perspective of the study?	Societal	Societal	HC payer	Societal	Societal	Societal	Societal	Societal	Societal	HC payer	HC Provider	HC provider
(b) Was the appropriate epidemiologic approach taken?												
(c) Was the study question well specified?												
(i) Were all relevant, non-trivial cost components and their stakeholders identified?												
(ii) Were necessary timeframes specified?												
(iii) Was a case of disease or risk factor adequately and appropriately defined?												
(iv) Was the counterfactual population occurrence plausible and meaningful?	NA		NA	NA	NA	NA	NA			NA	NA	NA
**2) Methodology and data: how well were resource use and productivity losses measured? (overall)**												
(a) Was an appropriate method(s) of quantification used, such that												
(i) additional, or excess costs were measured?												
(ii) only costs specific to (caused by) the health problem were included (confounders controlled)?												
(iii) all important effects were captured?												
(iv) important differences across subpopulations were accounted for?												
(v) the required level of detail could be provided?												
(b) Was the resource quantification method(s) well executed?												
(i) For population-based studies, were cost allocation methods, data and assumptions valid?<br>(ii) For person-based studies, were appropriate statistical tests performed and reported?												
(iii) Were data representative of the study population?												
(iv) Were there any other relevant resource quantification issues? (blue=no, orange=yes)												
(c) Were healthcare resources valued appropriately?												
(d) Was the approach for valuing production losses justified, and assumptions valid?											NA	NA
(e) Was the inclusion of intangible (indirect) costs appropriate:											NA	NA

**Question/Study**	Aajami, 2019	Allegri, 2007	Custodio, 2015	Ferretti, 2018	Jia, 2018	Keogh-Brown, 2015	Kongpak wattana, 2019	Koris, 2018	Liu, 2013	Mould-Quevedo, 2013	Nur, 2017	Prada, 2017
(i) Was double counting of mortality-related production losses avoided?	NA	NA	NA	NA	NA		NA	NA	NA	NA	NA	NA
(ii) Were losses valued appropriately, given the study's perspective?											NA	
**3) Analysis and reporting (overall)**												
(a) Did the analysis address the study question?												
(b) Was a range of estimates presented?												
(c) Were the main uncertainties identified?												
(d) Was a sensitivity analysis performed on:												
(i) important (uncertain) parameter estimates?												
(ii) key assumptions? (including the counterfactual)												
(iii) point estimates? (based on confidence or credible intervals)												
(e) Was adequate documentation and justification given for cost components, data, and sources, assumptions and methods?												
(f) Was uncertainty around the estimates and its implications adequately discussed?												
(g) Were important limitations discussed regarding the cost components, data, assumptions and methods?												
(h) Were the results presented at the appropriate level of detail to answer the study question (cost components; disease subtypes, severity, stage; subpopulation groups, cost bearers)?												

Blue box denotes the question/parameter was answered yes and appropriately.

Orange box denotes the question/parameter was answered no and not appropriately.

NA; Not applicable, HC: Healthcare

Continuation of Table 1...

**Question/Study**	Prince, 2004	Rao, 2013	Rojas, 2011	Wang, 2008	Wimo, 2006	Wimo, 2007	Wimo, 2010	Wimo, 2013	Wimo, 2017	Xu, 2017	Yan, 2019	Zencir, 2005
**1) Analytical framework: what costs should have been measured?**												
(a) What was the motivation												
and perspective of the study?	Societal	Societal	HC payer	Societal	HC payer	Societal	Societal	Societal	Societal	Societal	Societal	Societal
(b) Was the appropriate epidemiologic approach taken?												
(c) Was the study question well specified?												
(i) Were all relevant, non-trivial cost components and their stakeholders identified?												
(ii) Were necessary timeframes specified?												
(iii) Was a case of disease or risk factor adequately and appropriately defined?												
(iv) Was the counterfactual population occurrence plausible and meaningful?	NA	NA	NA	NA	NA	NA	NA	NA	NA	NA	NA	
**2) Methodology and data: how well were resource use and productivity losses measured?**												
(a) Was an appropriate method(s) of quantification used, such that												
(i) additional, or excess, costs were measured?												
(ii) only costs specific to (caused by) the health problem were included (confounders controlled)?												
(iii) all important effects were captured?												
(iv) important differences across subpopulations were accounted for?												
(v) the required level of detail could be provided?												
(b) Was the resource quantification method(s) well executed?												
(i) For population-based studies, were cost allocation methods, data and assumptions valid?<br>(ii) For person-based studies, were appropriate statistical tests performed and reported?												
(iii) Were data representative of the study population?												
(iv) Were there any other relevant resource quantification issues? (blue=no, orange=yes)												
(c) Were healthcare resources valued appropriately?												
(d) Was the approach for valuing production losses justified, and assumptions valid?			NA		NA							
(e) Was the inclusion of intangible (indirect) costs appropriate:			NA		NA							
(i) Was double counting of mortality-related production losses avoided?	NA	NA	NA	NA	NA	NA	NA	NA	NA	NA	NA	NA
(ii) Were losses valued appropriately, given the study's perspective?			NA		NA							

Question/Study	Prince, 2004	Rao, 2013	Rojas, 2011	Wang, 2008	Wimo, 2006	Wimo, 2007	Wimo, 2010	Wimo, 2013	Wimo, 2017	Xu, 2017	Yan, 2019	Zencir, 2005
**3) Analysis and reporting (overall)**												
(a) Did the analysis address the study question?												
(b) Was a range of estimates presented?												
(c) Were the main uncertainties identified?												
(d) Was a sensitivity analysis performed on:												
(i) important (uncertain) parameter estimates?												
(ii) key assumptions? (including the counterfactual)												
(iii) point estimates? (based on confidence or credible intervals)												
(e) Was adequate documentation and justification given for cost components, data and sources, assumptions and methods?												
(f) Was uncertainty around the estimates and its implications adequately discussed?												
(g) Were important limitations discussed regarding the cost components, data, assumptions and methods?												
(h) Were the results presented at the appropriate level of detail to answer the study question (cost components; disease subtypes, severity, stage; subpopulation groups, cost bearers)?												

Blue box denotes the question/parameter was answered yes and appropriately.
Orange box denotes the question/parameter was answered no and not appropriately.
NA; Not applicable, HC: Healthcare

Table 2. List of sources for the missing information to inflate national level data and obtaining cost of dementia as % of GDP.

Country	Missing data	Sources
Argentina	CPI	https://ycharts.com/indicators/argentina_consumer_price_index_wdi
Cambodia	CPI	World Bank 2018 CPI
Central Africa Republic	CPI	Chad's 2009 CPI (neighbouring country with the same World Bank's LMICs grouping)
Comoros	CPI	World Bank 2013 CPI
Congo	CPI	World Bank 2016 CPI
Cuba	Exchange rate	https://www.statista.com/outlook/998/203/economy/cuba#market-ExchangeRate
Eriteria	CPI	Ethiopia's CPI (neighbouring country with the same World Bank's LMICs grouping)
Gaza Strip	Exchange rate	Israel shekel World Bank
Libya	CPI	https://tradingeconomics.com/libya/consumer-price-index-cpi
Melanesia	CPI, Exchange rate, GDP	5 countries grouped under Melanesia: Fiji, Vanuatu, Solomon Islands, Papua New Guinea, New Caledonia, West New Guinea (indonesia). Only New Caledonia a high-income countries. We divide the cost report by the ratio of population reported by the countries, inflated the cost data and only took the average cost dementia as % GDP excluding New Caledonia data.
North Korea	GDP	http://data.un.org/CountryProfile.aspx/_Images/CountryProfile.aspx?crName=Democratic%20People%27s%20Republic%20of%20Korea
North Korea	Exchange rate	Frank, R. (2010). North Korea in 2009:: Domestic Developments and the Economy. In *Korea 2010* (pp. 29-53). Brill.
North Korea	CPI	No available data on CPI from North Korea, as the country's is practising closed economy and their economic practice are different than other country even in the same LMICs group, therefore we did not inflate them using the CPI inflation ratio.
Somalia	CPI	https://www.afdb.org/fr/documents/somalia-consumer-price-index-december-2019
Somalia	GDP	https://tradingeconomics.com/somalia/gdp GDP
Syria	CPI	World Bank 2012 CPI
Syria	Exchange rate	https://www.ceicdata.com/en/syria/foreign-Exchange-rates Exchange rate 2019
Syria	GDP	https://tradingeconomics.com/syria/gdp 2019 GDP
Timor Leste	CPI	World Bank 2018 CPI
Turkmenistan	Exchange rate, CPI	Iran's Exchange rate and CPI (neighbouring country with the same World Bank's LMICs grouping)
Uzbekistan	CPI	https://www.indexmundi.com/uzbekistan/inflation_rate_(consumer_prices).html
Uzbekistan	Exchange rate	https://fred.stlouisfed.org/series/FXRATEUZA618NUPN
Venezuela	CPI	https://ycharts.com/indicators/venezuela_consumer_price_index_wdi
Western Sahara	Exchange rate	Morroco's data (Western Sahara is a disputed area, they use Morrocco's currency)

Western Sahara	CPI	Morroco's data (Western Sahara is a disputed area, they use Morrocco's currency)
Western Sahara	GDP	Morroco's data (Western Sahara is a disputed area, they use Morrocco's currency)
Western Sahara	Population	http://data.un.org/CountryProfile.aspx/_Images/CountryProfile.aspx?crName=Western%20Sahara
Yemen	CPI	World Bank 2014 CPI
Zimbabwe	Exchange rate	https://knoema.com/atlas/Zimbabwe/topics/Economy/Short-term-indicators/Exchange-rate

Table 3. Direct cost components

**Author, year**	**Country**	**Medications (general)/ drugs**	**Hospital stays/ inpatient**	**Outpatient visits/ consultation/ emergency**	**Diagnostic, examination, and tests**	**Rehabilitation/ physical therapy**	**out-of-pocket**	**Other components**
Aajami, 2019	Iran	✓	×	✓	✓	✓	×	
Allegri, 2007	Argentina	✓	✓	✓	×	×	×	Ancillary studies
Custodio, 2015	Peru	✓	×	✓	✓	×	×	Antipsychotic drugs
Ferreti, 2018	Brazil	✓	✓	✓	×	×	×	
Jia, 2018	China	✓	✓	✓	×	×	×	
Keogh-Brown, 2015	China	✓	✓	✓	×	✓	×	
Kongpakwattana, 2019	Thailand	✓	✓	✓	×	×	✓	AD medications
Koris, 2018	Malaysia	✓	✓	✓	✓	×	×	Alternative treatment
Liu, 2013	China, Cuba, Dominican Republic, India, Mexico, Peru, Venezuela	✓	✓	×	×	×	×	Government primary care, hospital doctor, other government health worker, private doctor, dentist, traditional healer
Mould-Quevedo, 2013	China	✓	✓	✓	×	×	×	
Nur, 2017	Malaysia	✓	✓	✓	✓	✓	×	
Prada, 2017	Colombia	✓	✓	✓	×	×	✓	
Prince, 2004	Argentina, Brazil, Chile, Cuba, Dominican Republic, Guatemala, Mexico, Panama, Peru, Uruguay, Venezuela, China, India, Nigeria	×	✓	×	×	×	×	Government primary care, government hospital, health worker, private doctor, dentist, traditional healer

**Author, year**	**Country**	**Medications (general)/ drugs**	**Hospital stays/ inpatient**	**Outpatient visits/ consultation/ emergency**	**Diagnostic, examination, and tests**	**Rehabilitation/ physical therapy**	**out-of-pocket**	**Other components**
Rao, 2013	India	✓	✓	✓	✓	×	×	
Rojas, 2011	Argentina	×	✓	✓	×	×	×	Ancillary studies
Wang, 2008	China	✓	✓	✓	×	✓	×	
Wimo, 2006	Worldwide	×	×	×	×	×	×	
Wimo, 2007	Worldwide	×	×	×	×	×	×	
Wimo, 2010	Worldwide	×	×	×	×	×	×	
Wimo, 2013	Worldwide	×	×	×	×	×	×	
Wimo, 2017	Worldwide	×	×	×	×	×	×	
Xu, 2017	China	✓	✓	✓	✓	×	×	
Yan, 2019	China	✓	✓	✓	×	×	✓	
Zencir, 2005	Turkey	✓	×	✓	×	×	×	

Table 4. Direct non-medical cost components

Author, year	Country	Paid caregiver	Nursing home/ institutionalised/ accommodation	Transportations/ travel	Meals/ special food	Nappy	Equipment	Other components
Aajami, 2019	Iran	×	✓	✓	✓	×	✓	
Allegri, 2007	Argentina	✓	✓	×	×	×	×	
Custodio, 2015	Peru	✓	×	×	×	✓	×	
Ferreti, 2018	Brazil	×	×	✓	×	✓	×	
Jia, 2018	China	×	✓	✓	✓	×	×	
Keogh-Brown, 2015	China	✓	×	✓	×	×	✓	
Kongpakwattana, 2019	Thailand	✓	×	✓	×	×	×	
Koris, 2018	Malaysia	✓	×	✓	✓	×	×	
Liu, 2013	China, Cuba, Dominican Republic, India, Mexico, Peru, Venezuela	×	×	✓	×	×	×	
Mould-Quevedo, 2013	China	×	×	×	×	×	×	Housekeeper cost
Nur, 2017	Malaysia	Not applicable						
Prada, 2017	Colombia	Not applicable						
Prince, 2004	Argentina, Brazil, Chile, Cuba, Dominican Republic, Guatemala, Mexico, Panama, Peru, Uruguay, Venezuela, China, India, Nigeria	✓	×	×	×	×	×	
Rao, 2013	India	✓	✓	✓	✓	×	×	House modification
Rojas, 2011	Argentina	✓	×	×	×	×	×	
Wang, 2008	China	✓	×	✓	×	×	✓	

Author, year	Country	Paid caregiver	Nursing home/ institutionalised/ accommodation	Transportations/ travel	Meals/ special food	Nappy	Equipment	Other components
Wimo, 2006	Worldwide	×	×	×	×	×	×	
Wimo, 2007	Worldwide	×	×	×	×	×	×	
Wimo, 2010	Worldwide	×	×	×	×	×	×	
Wimo, 2013	Worldwide	×	×	×	×	×	×	
Wimo, 2017	Worldwide	×	×	×	×	×	×	
Xu, 2017	China	×	✓	✓	×	×	×	
Yan, 2019	China	×	✓	✓	✓	×	✓	
Zencir, 2005	Turkey	Not applicable						

Table 5. Indirect cost components reported by the included studies.

Author, year	Country	Productivity work loss of informal caregiver	Time spent on caregiving	Productivity work loss of patient	Health services for caregiver treatment	Other components
Aajami, 2019	Iran	✓	×	×	×	
Allegri, 2007	Argentina	×	✓	×	×	
Custodio, 2015	Peru	×	✓	×	×	
Ferreti, 2018	Brazil	✓	✓	×	✓	
Jia, 2018	China	✓	×	✓	✓	
Keogh-Brown, 2015	China	✓	✓	×	×	
Kongpakwattana, 2019	Thailand	×	✓	×	×	
Koris, 2018	Malaysia	×	✓	×	×	
Liu, 2013	China, Cuba, Dominican Republic, India, Mexico, Peru, Venezuela	×	✓	×	×	
Mould-Quevedo, 2013	China	✓	×	×	×	
Nur, 2017	Malaysia	Not applicable				
Prada, 2017	Colombia	Not applicable				
Prince, 2004	Argentina, Brazil, Chile, Cuba, Dominican Republic, Guatemala, Mexico, Panama, Peru, Uruguay, Venezuela, China, India, Nigeria	✓	×	×	×	
Rao, 2013	India	✓	✓	×	×	
Rojas, 2011	Argentina	×	×	×	×	
Wang, 2008	China	×	✓	×	×	
Wimo, 2006	Worldwide	Not applicable				

Author, year	Country	Productivity work loss of informal caregiver	Time spent on caregiving	Productivity work loss of patient	Health services for caregiver treatment	Other components
Wimo, 2007	Worldwide	×	✓	×	×	
Wimo, 2010	Worldwide	×	✓	×	×	
Wimo, 2013	Worldwide	×	✓	×	×	
Wimo, 2017	Worldwide	×	✓	×	×	
Xu, 2017	China	✓	✓	✓	×	
Yan, 2019	China	✓	×	✓	✓	
Zencir, 2005	Turkey	✓	✓	×	×	

Table 6. The annual national cost of dementia as percentage of GDP of LMICs or region, inflated to year 2019.

Study	Country	GDP 2019	Direct Cost	Indirect Cost	Total Cost	Direct Cost as % of GDP	Indirect Cost as % of GDP	Total Cost as % of GDP
**UMIC**								
Wimo, 2010	Albania	15,279,183,290	51,409,966	26,558,261	77,968,227	0.34	0.17	0.51
Wimo, 2010	Algeria	171,091,289,782	319,088,207	104,065,071	423,153,278	0.19	0.06	0.25
Wimo, 2010	Argentina	445,445,177,459	544,810,220	368,902,641	913,712,862	0.12	0.08	0.21
Wimo, 2010	Armenia	13,672,802,158	55,155,186	23,562,464	78,717,650	0.40	0.17	0.58
Wimo, 2010	Azerbaijan	48,047,647,059	82,119,058	53,177,384	135,296,442	0.17	0.11	0.28
Wimo, 2010	Belarus	63,080,457,023	325,403,611	146,435,280	471,838,891	0.52	0.23	0.75
Wimo, 2010	Belize	1,879,613,600	3,552,335	2,925,452	6,477,787	0.19	0.16	0.34
Wimo, 2010	Bosnia and Herzegovina	20,164,193,807	80,613,887	133,724,212	214,338,098	0.40	0.66	1.06
Wimo, 2010	Botswana	18,340,480,936	27,275,114	14,702,991	41,978,105	0.15	0.08	0.23
Wimo, 2010	Brazil	1,839,758,040,766	3,691,344,114	3,346,416,992	7,037,761,106	0.20	0.18	0.38
Wimo, 2010	Bulgaria	68,558,815,112	444,034,193	194,129,295	638,069,113	0.65	0.28	0.93
Jia, 2018	China	14,343,000,000,000	93,915,262,557	101,220,946,303	195,136,208,860	0.65	0.71	1.36
Wimo, 2010	Colombia	323,615,979,419	628,902,071	529,427,060	1,158,329,130	0.19	0.16	0.36
Wimo, 2010	Costa Rica	61,801,385,049	198,943,971	124,124,028	323,067,999	0.32	0.20	0.52

Wimo, 2007	Cuba	95,150,000,000	241,734,580	177,686,811	419,421,391	0.25	0.19	0.44
Wimo, 2007	Dominican Republic	88,941,299,734	82,736,978	169,616,254	252,353,232	0.09	0.19	0.28
Wimo, 2010	Ecuador	107,435,665,000	179,205,912	187,561,999	366,767,911	0.17	0.17	0.34
Wimo, 2010	Equatorial Guinea	11,026,774,945	10,220,474	4,733,693	14,954,168	0.09	0.04	0.14
Wimo, 2010	Gabon	16,874,450,215	13,781,863	13,282,520	27,064,383	0.08	0.08	0.16
Wimo, 2010	Georgia	17,477,255,546	68,628,742	39,955,671	108,584,413	0.39	0.23	0.62
Wimo, 2010	Guatemala	76,710,355,347	144,495,613	165,272,760	309,768,373	0.19	0.22	0.40
Wimo, 2010	Guayana	5,173,759,528	8,960,330	9,549,826	18,510,156	0.17	0.18	0.36
Wimo, 2010	Iran	583,700,000,000	1,963,370,101	1,177,623,204	3,140,850,856	0.34	0.20	0.54
Wimo, 2010	Iraq	234,094,042,939	98,115,369	457,481,532	555,596,901	0.04	0.20	0.24
Wimo, 2010	Jordan	44,502,895,862	51,095,785	50,832,405	101,928,190	0.11	0.11	0.23
Wimo, 2010	Kazakhstan	181,665,908,556	146,372,178	69,049,572	215,421,750	0.08	0.04	0.12
Wimo, 2010	Lebanon	51,991,634,492	83,420,939	69,089,303	152,510,242	0.16	0.13	0.29
Wimo, 2010	Libya	52,091,152,228	237,089,261	139,391,709	376,234,259	0.46	0.27	0.72
Koris, 2018	Malaysia	364,681,367,532	212,462,032	1,912,158,302	2,124,620,335	0.06	0.52	0.58
Wimo, 2010	Maldives	5,642,179,170	2,404,762	1,311,688	3,716,450	0.04	0.02	0.07
Wimo, 2010	Mauritius	14,048,430,519	54,341,314	31,448,078	85,909,883	0.39	0.22	0.61
Wimo, 2010	Mexico	1,268,870,527,160	2,668,227,175	1,108,141,635	3,776,368,810	0.21	0.09	0.30

Wimo, 2010	Montenegro	5,542,577,965	9,344,893	6,031,704	15,461,550	0.17	0.11	0.28
Wimo, 2010	Namibia	12,366,528,259	18,469,639	5,863,377	24,235,293	0.15	0.05	0.20
Wimo, 2010	Paraguay	38,145,288,940	71,870,270	71,630,702	143,500,972	0.19	0.19	0.38
Wimo, 2010	Peru	226,848,050,820	383,114,880	331,866,427	714,981,307	0.17	0.15	0.32
Wimo, 2010	Russia	1,699,876,578,871	8,602,715,233	3,714,703,400	12,317,418,633	0.51	0.22	0.72
Wimo, 2010	Serbia	51,475,016,532	314,750,429	203,615,842	518,366,271	0.61	0.40	1.01
Wimo, 2010	South Africa	351,431,649,241	815,632,235	1,306,330,710	2,121,962,944	0.23	0.37	0.60
Wimo, 2010	Sri Lanka	84,008,783,756	328,369,104	12,323,137	446,926,180	0.39	0.01	0.53
Wimo, 2010	Suriname	3,697,371,950	8,234,763	11,250,310	19,485,073	0.22	0.30	0.53
Wimo, 2010	Thailand	543,548,969,969	1,546,075,473	264,609,810	818,237,070	0.28	0.05	0.15
Wimo, 2010	North Macedonia	12,547,040,499	50,073,021	25,406,053	75,479,074	0.40	0.20	0.60
Wimo, 2010	Turkey	761,425,499,358	794,509,615	724,354,701	1,518,864,316	0.10	0.10	0.20
Wimo, 2010	Turkmenistan	40,761,000,000	86,893,692	46,580,717	133,474,409	0.21	0.11	0.33
Wimo, 2010	Venezuela	202,010,000,000	479,262	744,944	1,224,206	0.00	0.00	0.00
Wimo, 2010	Fiji	5,496,250,694	724,579	3,530,214	4,254,792	0.01	0.06	0.08
**LRMIC**								
Wimo, 2010	Angola	88,815,697,793	41,494,010	31,120,508	72,614,518	0.05	0.04	0.08
Wimo, 2010	Bangladesh	302,571,254,131	591,927,746	704,214,550	1,295,983,475	0.20	0.23	0.43

Mattap SM, *et al. BMJ Global Health* 2022; 7:e007409. doi: 10.1136/bmjgh-2021-007409

Wimo, 2010	Bhutan	2,530,547,158	4,428,117	3,075,082	7,503,199	0.17	0.12	0.30
Wimo, 2010	Bolivia	40,895,322,844	63,942,499	131,591,809	195,534,307	0.16	0.32	0.48
Wimo, 2010	Cambodia	27,089,389,787	51,531,731	64,921,865	116,453,596	0.19	0.24	0.43
Wimo, 2010	Cameroon	39,007,354,359	43,016,607	37,615,419	80,632,026	0.11	0.10	0.21
Wimo, 2010	Cape verde	1,981,845,740	3,849,640	1,283,213	5,132,853	0.19	0.06	0.26
Wimo, 2010	Comoros	1,165,839,927	943,527	943,527	1,887,054	0.08	0.08	0.16
Wimo, 2010	Congo	12,267,392,147	5,427,916	8,443,425	13,871,341	0.04	0.07	0.11
Wimo, 2010	Cote d'Ivoire	58,539,424,930	26,569,920	76,717,243	103,377,845	0.05	0.13	0.18
Wimo, 2010	Democratic Republic of the Congo	50,400,746,171	39,819,605	24,244,149	64,134,231	0.08	0.05	0.13
Wimo, 2010	Djibouti	3,324,634,256	2,250,029	2,000,026	4,250,055	0.07	0.06	0.13
Wimo, 2010	East Timor/Timor Leste	2,017,924,900	2,327,877	3,569,412	5,897,289	0.12	0.18	0.29
Wimo, 2010	Egypt	303,092,255,125	216,737,320	186,623,354	403,360,674	0.07	0.06	0.13
Wimo, 2010	El Salvador	27,022,640,000	88,006,364	66,173,584	154,292,488	0.33	0.24	0.57
Wimo, 2010	Gaza Strip	16,277,000,000	3,742,117	16,493,036	20,235,154	0.02	0.10	0.12
Wimo, 2010	Ghana	66,983,634,224	70,323,423	142,566,940	212,890,363	0.10	0.21	0.32
Wimo, 2010	Honduras	25,095,395,475	46,722,031	73,420,335	120,021,011	0.19	0.29	0.48
Rao, 2013	India	2,875,000,000,000	3,238,636,795	3,455,906,311	6,694,543,106	0.11	0.12	0.23
Wimo, 2010	Indonesia	1,119,190,780,753	2,206,416,654	1,818,312,365	4,024,729,018	0.20	0.16	0.36

Wimo, 2010	Kenya	95,503,088,538	49,933,369	73,056,876	122,906,464	0.05	0.08	0.13
Wimo, 2010	Kyrgyzstan	8,454,619,608	25,625,801	26,452,439	52,078,240	0.30	0.31	0.62
Wimo, 2010	Laos	18,173,839,147	19,345,273	29,794,544	49,281,023	0.11	0.16	0.27
Wimo, 2010	Lesotho	2,376,328,637	6,619,996	58,634,254	12,483,422	0.28	2.47	0.53
Wimo, 2010	Mauretania	7,600,656,944	5,335,973	10,774,561	16,110,534	0.07	0.14	0.21
Wimo, 2010	Micronesia	410,000,000	495,634	758,028	1,253,662	0.12	0.18	0.31
Wimo, 2010	Mongolia	13,996,719,329	10,466,115	13,489,659	23,955,774	0.07	0.10	0.17
Wimo, 2010	Morocco	119,700,325,462	191,746,560	178,312,087	370,058,647	0.16	0.15	0.31
Wimo, 2010	Myanmar	76,085,852,617	1,871,748	381,935	2,253,018	0.00	0.00	0.00
Wimo, 2010	Nicaragua	12,520,915,291	43,735,668	62,494,581	106,230,248	0.35	0.50	0.85
Wimo, 2010	Nigeria	448,120,428,859	256,766,981	1,210,789,128	1,467,703,676	0.06	0.27	0.33
Wimo, 2010	Pakistan	278,221,906,023	835,693,815	884,476,264	1,720,170,079	0.30	0.32	0.62
Wimo, 2010	Philippines	376,795,508,679	653,600,908	397,881,720	1,051,606,463	0.17	0.11	0.28
Wimo, 2010	Republic of Moldova	11,968,713,370	42,574,135	3,613,720	46,187,855	0.36	0.03	0.39
Wimo, 2010	Senegal	23,578,084,052	9,320,874	22,281,326	31,602,200	0.04	0.09	0.13
Wimo, 2010	Sudan	30,513,456,085	184,317,780	147,598,222	331,916,002	0.60	0.48	1.09
Wimo, 2010	Swaziland/Eswatini	4,471,598,727	6,031,284	12,369,244	18,400,529	0.13	0.28	0.41
Wimo, 2010	Tunisia	38,796,687,568	126,268,408	36,023,851	162,292,259	0.33	0.09	0.42

Wimo, 2010	Ukraine	153,781,069,118	2,418,024,208	1,650,214,694	4,068,238,902	1.57	1.07	2.65
Wimo, 2010	Uzbekistan	57,921,286,440	24,131,459	34,849,186	58,980,645	0.04	0.06	0.10
Wimo, 2010	Viet Nam	261,921,244,843	973,293,555	701,083,364	2,003,831,896	0.37	0.27	0.77
Wimo, 2010	Western Sahara (Morocco/Mauritania)	119,700,325,462	93,947	1,221,316	1,315,263	0.00	0.00	0.00
Wimo, 2010	Zambia	23,309,773,923	11,078,665	28,462,261	39,450,856	0.05	0.12	0.17
Wimo, 2010	Zimbabwe	21,440,758,800	14,124,603	38,259,435	52,384,038	0.07	0.18	0.24
Wimo, 2011	Vanuatu	934,239,974	176,878	861,767	1,038,646	0.02	0.09	0.11
Wimo, 2012	Solomon Islands	1,589,913,622	466,568	2,273,164	2,739,732	0.03	0.14	0.17
Wimo, 2013	Papua New Guinea	24,829,107,011	6,452,060	31,435,038	37,887,098	0.03	0.13	0.15
Wimo, 2014	Western New Guinea (Indonesia)	1,119,190,780,753	2,866,849	13,967,555	16,834,404	0.000	0.001	0.002
**LIC**								
Wimo, 2010	Afghanistan	19,291,104,008	22,110,839	58,202,074	80,312,913	0.11	0.30	0.42
Wimo, 2010	Benin	14,390,709,095	7,628,770	28,607,888	36,236,658	0.05	0.20	0.25
Wimo, 2010	Burkina Faso	15,990,803,570	7,595,445	25,807,251	33,402,697	0.05	0.16	0.21
Wimo, 2010	Burundi	3,012,334,882	8,014,552	4,524,344	12,538,895	0.27	0.15	0.42
Wimo, 2010	Central African Republic	2,220,307,369	16,865,565	25,947,023	42,812,588	0.76	1.17	1.93
Wimo, 2010	Chad	11,314,951,343	10,546,064	12,211,232	22,757,296	0.09	0.11	0.20

Wimo, 2010	Democratic People's Republic of Korea	17,396,000,000	16,278,770	53,975,922	70,254,692	0.09	0.31	0.40
Wimo, 2010	Eritrea	6,500,000,000	13,022,511	27,100,901	40,123,412	0.20	0.42	0.62
Wimo, 2010	Ethiopia	95,912,590,628	147,623,436	70,316,845	217,940,281	0.15	0.07	0.23
Wimo, 2010	Gambia	1,826,073,740	1,834,891	5,118,381	6,953,272	0.10	0.28	0.38
Wimo, 2010	Guinea	12,296,665,331	69,404,565	217,666,646	287,319,973	0.56	1.77	2.34
Wimo, 2010	Guinea-Bissau	1,339,449,165	776,585	1,466,883	2,243,468	0.06	0.11	0.17
Wimo, 2007	Haiti	14,332,163,266	18,835,225	59,854,160	78,689,385	0.13	0.42	0.55
Wimo, 2010	Liberia	3,070,518,100	2,838,633	123,480,551	15,186,688	0.09	4.02	0.49
Wimo, 2010	Madagascar	14,114,631,281	20,248,451	51,709,754	72,067,068	0.14	0.37	0.51
Wimo, 2010	Malawi	7,666,704,427	7,572,626	5,445,484	13,018,110	0.10	0.07	0.17
Wimo, 2010	Mali	17,279,566,719	6,441,445	23,648,046	30,001,253	0.04	0.14	0.17
Wimo, 2010	Mozambique	15,291,448,212	29,037,810	31,382,478	60,420,288	0.19	0.21	0.40
Wimo, 2010	Nepal	30,641,380,604	84,997,386	116,693,735	201,691,121	0.28	0.38	0.66
Wimo, 2010	Niger	12,911,689,659	4,953,562	29,013,721	33,967,284	0.04	0.22	0.26
Wimo, 2010	Rwanda	10,354,417,725	11,807,671	13,236,019	24,948,467	0.11	0.13	0.24
Wimo, 2010	Sierra Leone	4,121,733,705	2,263,585	14,996,249	17,259,834	0.05	0.36	0.42
Wimo, 2010	Somalia	7,700,000,000	7,960,785	22,866,086	30,996,250	0.10	0.30	0.40
Wimo, 2010	Syrian Arab Republic	65,000,000,000	3,567,655	4,924,748	8,496,247	0.01	0.01	0.01

Wimo, 2010	Tajikistan	8,116,626,794	7,123,631	12,431,435	19,601,626	0.09	0.15	0.24
Wimo, 2010	Togo	5,490,272,413	9,040,993	23,894,054	32,935,047	0.16	0.44	0.60
Wimo, 2010	Uganda	35,165,157,017	40,533,169	38,848,416	79,480,688	0.12	0.11	0.23
Wimo, 2010	United Republic of Tanzania	63,177,068,175	35,604,620	67,878,485	103,483,105	0.06	0.11	0.16
Wimo, 2010	Yemen	22,581,081,994	20,735,253	67,460,585	88,195,838	0.09	0.30	0.39
